# SARS-CoV-2 infection induces persistent adipose tissue damage in aged golden Syrian hamsters

**DOI:** 10.1038/s41419-023-05574-w

**Published:** 2023-02-01

**Authors:** Gemma Bogard, Johanna Barthelemy, Aline Hantute-Ghesquier, Valentin Sencio, Patricia Brito-Rodrigues, Karin Séron, Cyril Robil, Anne Flourens, Florence Pinet, Delphine Eberlé, François Trottein, Martine Duterque-Coquillaud, Isabelle Wolowczuk

**Affiliations:** 1 Univ. Lille, Institut National de la Santé et de la Recherche Médicale (Inserm), Centre National de la Recherche Scientifique (CNRS), Centre Hospitalier Universitaire de Lille (CHU Lille), Institut Pasteur de Lille, U1019–UMR9017—Center for Infection and Immunity of Lille (CIIL), F-59000 Lille, France; 2 Univ. Lille, CNRS, Inserm, CHU Lille, Institut Pasteur de Lille, UMR9020–U1277–CANTHER–Cancer Heterogeneity Plasticity and Resistance to Therapies, F-59000 Lille, France; 3 Univ. Lille, Inserm, CHU Lille, Institut Pasteur de Lille, U1167–RID-AGE–Facteurs de risque et déterminants moléculaires des maladies liées au vieillissement, F-59000 Lille, France; 4 Univ. Lille, Inserm, CHU Lille, Institut Pasteur de Lille, U1011–EGID, F-59000 Lille, France

**Keywords:** Infection, Ageing

## Abstract

Coronavirus disease 2019 (COVID-19, caused by severe acute respiratory syndrome-coronavirus 2 (SARS-CoV-2)) is primarily a respiratory illness. However, various extrapulmonary manifestations have been reported in patients with severe forms of COVID-19. Notably, SARS-CoV-2 was shown to directly trigger white adipose tissue (WAT) dysfunction, which in turn drives insulin resistance, dyslipidemia, and other adverse outcomes in patients with COVID-19. Although advanced age is the greatest risk factor for COVID-19 severity, published data on the impact of SARS-CoV-2 infection on WAT in aged individuals are scarce. Here, we characterized the response of subcutaneous and visceral WAT depots to SARS-CoV-2 infection in young adult and aged golden hamsters. In both age groups, infection was associated with a decrease in adipocyte size in the two WAT depots; this effect was partly due to changes in tissue’s lipid metabolism and persisted for longer in aged hamsters than in young-adult hamsters. In contrast, only the subcutaneous WAT depot contained crown-like structures (CLSs) in which dead adipocytes were surrounded by SARS-CoV-2-infected macrophages, some of them forming syncytial multinucleated cells. Importantly, older age predisposed to a unique manifestation of viral disease in the subcutaneous WAT depot during SARS-CoV-2 infection; the persistence of very large CLSs was indicative of an age-associated defect in the clearance of dead adipocytes by macrophages. Moreover, we uncovered age-related differences in plasma lipid profiles during SARS-CoV-2 infection. These data suggest that the WAT’s abnormal response to SARS-CoV-2 infection may contribute to the greater severity of COVID-19 observed in elderly patients.

## Introduction

The continuing pandemic of coronavirus disease 2019 (COVID-19, caused by infection with the severe acute respiratory syndrome-coronavirus 2 (SARS-CoV-2)) has emphasized the urgent need to identify the disease’s pathophysiological mechanisms. COVID-19 features a broad spectrum of manifestations, ranging from asymptomatic disease to acute respiratory distress syndrome and potentially fatal multi-organ dysfunction [1–3]. The outcomes and severity of SARS-CoV-2 infections in golden (Syrian) hamsters resemble those observed in human COVID-19; hence, the golden hamster model is of relevance in the study of SARS-CoV-2 infection and COVID-19 pathogenesis [4–10].

Population-based cohort studies in humans and preclinical studies in golden hamsters have shown that obesity and aging are major independent risk factors for severe COVID-19 [11–17]. Obesity and aging have some phenotypic features in common, such as enhanced systemic inflammation [18, 19] and profound changes in white adipose tissue (WAT) distribution and function [20]. Studies in the context of obesity have shown that SARS-CoV-2 directly triggers a pathogenic inflammation in WAT; in turn this inflammation contributes to the cytokine storm associated with severe COVID-19 [21, 22]. In contrast, few studies have investigated the pathophysiology of WAT during SARS-CoV-2 infection in aged individuals.

The main function of WAT is to collect, store and release energy in the form of lipids, in response to systemic nutritional and metabolic needs. WAT is comprised of large, lipid-filled adipocytes and a heterogenous population of stromal vascular cells, which includes adipocyte precursors (preadipocytes), endothelial cells, stem/stromal cells, and immune cells [23, 24]. Based on the anatomical location, WAT can be subdivided into two main types of depot: subcutaneous and visceral [25, 26]. It has been shown that subcutaneous adipose tissue (SCAT) and visceral adipose tissue (VAT) differ significantly with regard to their cellular, molecular, and physiological characteristics [27, 28]. While excess VAT is commonly associated with metabolic disorders [29], SCAT appears to preserve metabolic health [30]. During aging, WAT undergoes profound changes in quantity, distribution, cellular composition and function; the main change is greater visceral adiposity, which increases the likelihood of age-associated metabolic disorders [27, 31, 32].

Although SARS-CoV-2 primarily targets the respiratory system, many other organ systems (including WAT) are affected during infection [21, 33–39]. Reiterer et al. were the first to suggest that SARS-CoV-2 triggers WAT dysfunction, which in turn contributes to adverse COVID-19 outcomes [35]. Indeed, these investigators reported that human adipocytes infected with SARS-CoV-2 in vitro produced lower amounts of adiponectin—an insulin-sensitizing, anti-inflammatory adipokine [40]. Importantly, Reiterer et al. detected viral RNA and low adiponectin expression in WAT from infected golden hamsters. These changes were associated with a robust inflammatory antiviral response in WAT and a systemic insulin-resistant state, suggesting that hyperglycemia in severe COVID-19 might result (at least in part) from infection-induced WAT dysfunction [35]. Martinez-Colon et al. identified mature, lipid-laden adipocytes and macrophages as the two main cellular targets of SARS-CoV-2 in human WAT [21]. Strikingly, preadipocytes are not permissive to infection—confirming that lipid-droplet metabolism is critical for SARS-CoV-2 propagation [41]. Most recently, SARS-CoV-2’s ability to infect mature adipocytes was confirmed: infected adipocytes are less viable and have a smaller lipid-droplet size and a higher prevalence of pyknotic nuclei, which are suggestive of infection-induced cell delipidation and death [42].

The impact of SARS-CoV-2 infection on the WAT of aged individuals has not previously been characterized. To address this knowledge gap, we compared the effects of SARS-CoV-2 infection on SCAT and VAT depots in young adult vs. aged golden hamsters. We found that SARS-CoV-2 infection impairs lipid metabolism in both SCAT and VAT, irrespective of the host’s age. In striking contrast, viral infection induced adipocyte death (detected as crown-like structures (CLSs)) in the SCAT but not in the VAT. Importantly, aging was associated with the impaired clearance of dead adipocytes in the SCAT likely due to the compromised efferocytosis capacity of tissue’s macrophages. These SARS-CoV-2-infection-induced changes in WAT were associated with age-specific blood lipid signatures. We hypothesize that these features might contribute to the severity of COVID-19 in the elderly.

## Results

### SARS-CoV-2 infection is more severe in aged golden hamsters than in young-adult golden hamsters

**Fig. 1:**
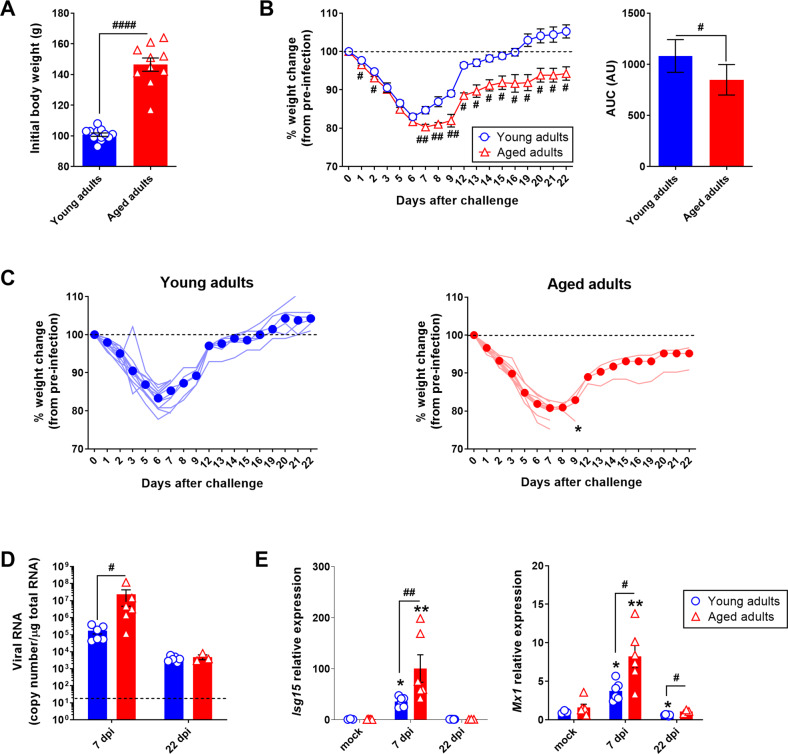
SARS-CoV-2 infection is more severe in aged golden hamsters. Golden (Syrian) hamsters aged 2 months (the “young adults” group) or 22 months (the “aged adults” group) were treated intranasally with a SARS-CoV-2 inoculate (*n* = 12 young adults and *n* = 10 aged adults) or with DMEM (mock infection) (*n* = 6 young adults and *n* = 6 aged adults). **A** Body weight (g) on the day of infection. Data are expressed as the mean ± SEM, and individual replicates are shown (*n* = 12 young adults and *n* = 10 aged adults). **B** Percentage body weight change after infection (left). Data are expressed as the mean ± SEM (*n* = 12 young adults and *n* = 10 aged adults). The corresponding area under the curve (AUC) is shown (right). Data are expressed in arbitrary units (AU). **C** Percentage body weight change after infection in individual animals. Median weight losses are depicted as plain circles. Asterisks indicate death. **D** Quantification of SARS-CoV-2 RNA in the lungs of young adult and aged adult hamsters, using an RT-qPCR assay. Viral RNA levels are expressed as the mean ± SEM copy number/μg of total RNA, and individual replicates are shown (mock and 7 days post infection (dpi): *n* = 6 animals per group, 22 dpi: *n* = 6 young adults and *n* = 3 aged adults). The dashed line represents the assay’s limit of detection. **E** mRNA expression levels (RT-qPCR assay) of the *Isg15* and *Mx1* genes in the lungs of young adult and aged adult hamsters, presented according to the 2^−ΔΔCT^ method (housekeeping gene: *Actg1*, coding for gamma (γ) actin). Data are expressed as the mean ± SEM, and individual replicates are shown (mock and 7 dpi: *n* = 6 animals per group, 22 dpi: *n* = 6 young adults and *n* = 3 aged adults). Groups were compared in a two-sided Mann–Whitney test; # indicates the *P* values for the comparison of young adults and aged adults (the effect of age: ^#^*P* < 0.05, ^##^*P* < 0.01 and ^####^*P* < 0.0001), and * indicates the *P* values for the comparison of mock-treated and SARS-CoV-2-infected groups (the effect of infection: *P < 0.05 and **P < 0.01). For intergroup differences, the threshold for statistical significance was set to *P* < 0.05.

Young adult male golden hamsters (2 months of age, the “young adults” group) and aged adult male golden hamsters (22 months of age, the “aged adults” group) were inoculated intranasally with a sublethal dose (2 × 10^4^ TCID_50_) of a clinical SARS-CoV-2 isolate or with DMEM (mock) (Supplementary Fig. S1). As reported previously [43], aging was associated with significant body weight gain; on average, aged hamsters were 45% heavier than young-adult hamsters (Fig. 1A). In both young-adult and aged hamsters, SARS-CoV-2 infection induced moderate body weight loss; this started on day 1 post infection (1 dpi) and changed to the same extent in both groups until 6 dpi (mean ± SEM percentage change from starting weight: 83 ± 2.7% for young adults, *P* < 0.0001, and 82 ± 2.3% for aged adults, *P* < 0.0001). However, from 6 dpi onward, the change in body weight diverged in the two groups. Young adults grew steadily heavier and had returned to their baseline weight by 15 dpi. In contrast, aged hamsters continued to lose weight until 7 dpi (80 ± 2.3% change from starting weight, *P* < 0.0001), after which time the body weight increased but at a much slower rate than in younger animals; at 22 dpi, aged hamsters had not recovered their initial body weight (94 ± 3% of the starting weight, *P* < 0.05) (Fig. 1B). Six young adult or aged SARS-CoV-2-infected animals were necropsied at 7 dpi, and the remaining animals (six young adult and four aged hamsters) were followed up until 22 dpi. One of the four remaining animals in the aged group lost a large amount of weight and suffered from respiratory distress at 9 dpi and so was sacrificed (Fig. 1C). We next quantified the SARS-CoV-2 RNA copy number in the lungs by measuring the expression of the *E* gene that encodes the viral envelope protein (Fig. 1D). Viral RNA levels were substantially higher in the aged group than in the young-adult group at 7 dpi but were lower and similar in the two groups at 22 dpi. In line with these findings, the expression levels of the interferon (IFN)-stimulated genes (ISGs) *Isg15* and IFN-induced GTP-binding protein *Mx1* were significantly higher in the lungs of aged animals at 7 dpi (Fig. 1E).

Relative to young-adult animals, aged hamsters displayed a higher pulmonary viral burden and a stronger early antiviral response (at 7 dpi) and failed to recover their initial body weight at later timepoints (at 22 dpi). Overall, these results suggest that aging weakens the whole-body metabolic response to SARS-CoV-2 infection and contributes to long-term sequelae.

### In both young adult and aged golden hamsters, SARS-CoV-2 infection leads to a lasting reduction in adipocyte size in SCAT and VAT

We next looked at the effects of SARS-CoV-2 infection on the two main WAT depots i.e., the inguinal SCAT and the epididymal VAT.

In accordance with their higher initial body weight (see Fig. 1A), aged hamsters displayed greater absolute SCAT and VAT masses than young adults did (Supplementary Fig. S2A). Nevertheless, when the SCAT and VAT masses were expressed as a percentage of body weight, only VAT was found to be more abundant in aged hamsters (Supplementary Fig. S2B).

SARS-CoV-2 infection was associated with markedly lower absolute and relative SCAT and VAT masses at 7 dpi in both age groups. However, at 22 dpi, only the aged animals still presented low absolute SCAT and VAT masses (Supplementary Fig. S2A), in line with their change in body weight (see Fig. 1B). Importantly, only aged hamsters displayed low relative SCAT mass at 22 dpi (Supplementary Fig. S2B); this might indicate regional differences in the WAT’s response to SARS-CoV-2 infection, with perhaps a greater impact on SCAT than on VAT.

We next used quantitative histomorphometry to assess the age-dependent effects of SARS-CoV-2 infection on adipocyte cell size and size distribution in SCAT and VAT at 0 (mock), 7, and 22 dpi.

**Fig. 2:**
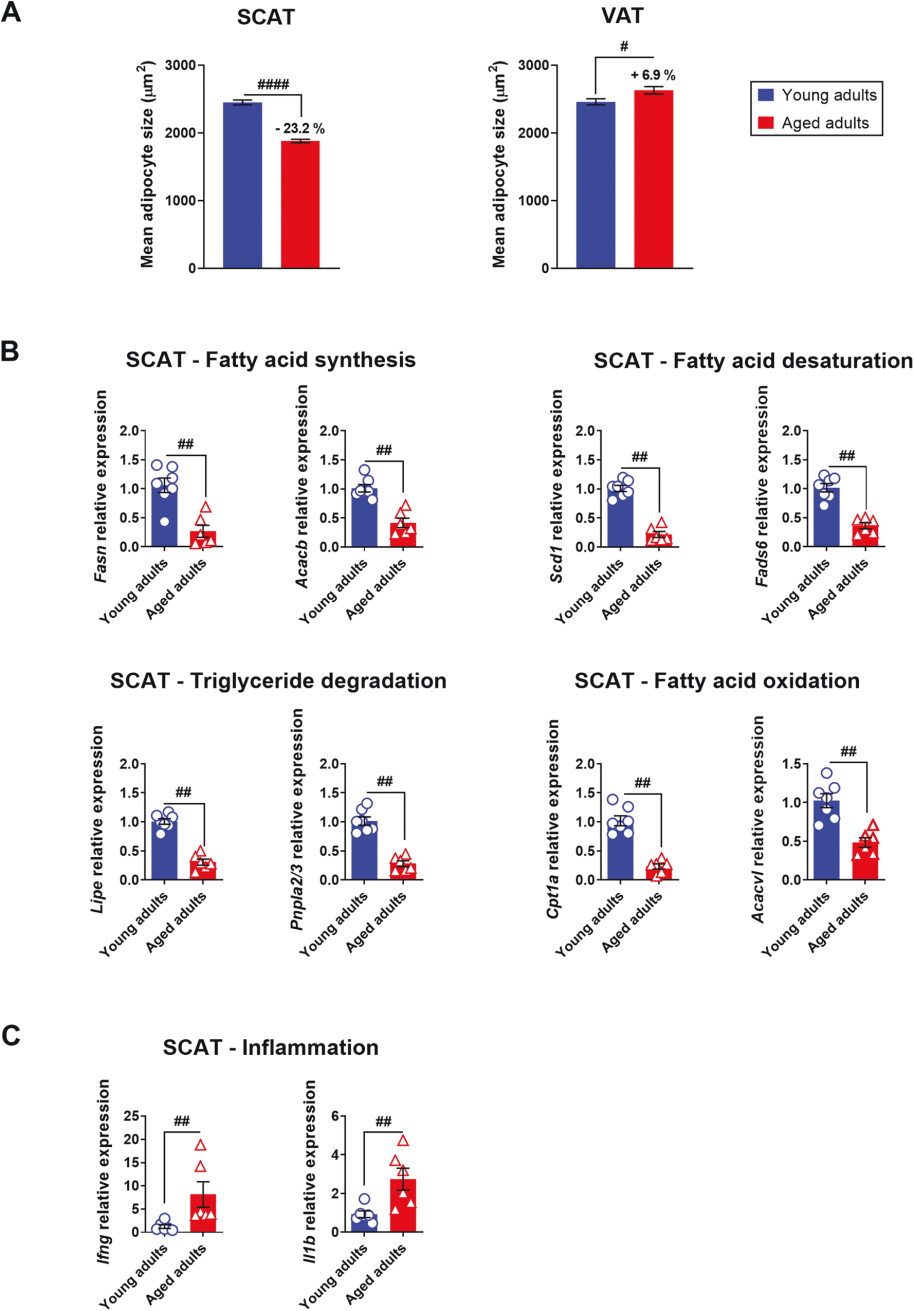
Aging has a regional impact on WAT in golden hamsters. **A** Mean ± SEM adipocyte size (μm^2^) in the (inguinal) SCAT and (epididymal) VAT of mock-treated young adult and aged hamsters. For SCAT, 2052 adipocytes in young adults and 2621 in aged adults were measured. For VAT, 1531 adipocytes in young adults and 1412 in aged adults were measured. Percentage differences in adipocyte size between young adult and aged hamsters are indicated. **B** mRNA expression levels (RT-qPCR assay) of *Fasn* and *Acacb* (involved in fatty acid synthesis), *Scd1* and *Fasd6* (involved in fatty acid desaturation), *Lipe* and *Pnpla2/3* (involved in triglyceride degradation), and *Cpt1a* and *Acadvl* (involved in fatty acid oxidation) in the SCAT of mock-treated young adult and aged hamsters. **C** mRNA expression levels (RT-qPCR assay) of the inflammatory genes *Ifng* and *Il1b* in the SCAT of mock-treated young adult and aged hamsters. **B**, **C** Relative expression is presented as 2^−ΔΔCT^ (housekeeping gene: *GusB*, coding for glucuronidase beta). Data are expressed as the mean ± SEM, and individual replicates are shown (*n* = 6 animals per group). Groups were compared in a two-sided Mann–Whitney test; ^#^ indicates the *P* values for the comparison of young adults and aged adults (the effect of age: ^#^*P* < 0.05, ^##^*P* < 0.01 and ^####^*P* < 0.0001). For intergroup differences, the threshold for statistical significance was set to *P* < 0.05.

First, our analysis of samples from mock-treated animals revealed that aging was associated with low mean adipocyte size in SCAT (a 23.2% difference between young adults and aged adults, *P* < 0.0001), and slight but significant high mean adipocyte size in VAT (a 6.9% difference between young adults and aged adults, *P* < 0.05) (Tables 1 and 2 and Fig. 2A). Changes in adipocyte size can reflect increased accumulation or release of stored lipids through lipogenesis (fatty acid synthesis and fatty acid desaturation) and lipolysis (triglyceride degradation and fatty acid oxidation), respectively. We therefore compared the expression levels of lipogenic genes (*Fasn*, encoding fatty acid synthase, *Acacb*, encoding acetyl-CoA carboxylase beta, *Scd1*, encoding stearoyl-CoA desaturase 1, and *Fads6*, encoding fatty acid desaturase 6) and lipolytic genes (*Lipe*, encoding hormone-sensitive lipase E, *Pnpla2/3*, encoding adipose triglyceride lipase, *Cpt1a*, encoding carnitine palmitoyl transferase 1A, and *Acadvl*, encoding very long-chain acyl-CoA dehydrogenase) in the SCAT (Fig. 2B) and the VAT (Supplementary Fig. S3A) from mock-treated young adult and aged hamsters. Compared with samples from young-adult hamsters, the mRNA expression levels of genes involved in the regulation of adipocyte lipid metabolism were significantly lower for the SCAT from aged animals (Fig. 2B) but were similar in the two age groups for the VAT (Supplementary Fig. S3A). Accordingly, the expression levels of *Ifng* and *Il1b*—which encode inflammatory cytokines critically involved in the regulation of lipid metabolism [44]—were significantly higher in the SCAT samples from aged animals (Fig. 2C) but not in the VAT samples (Supplementary Fig. S3B). These results show that in the golden hamster, aging per se affects adipocyte lipid metabolism and triggers inflammation (a phenomenon known as inflammaging [45–48]) in the SCAT but not in the VAT.

**Table 1:** Adipocyte size frequency distribution in SCAT.

	Young adults			Aged adults		
	mock	Day 7	Day 22	Mock	Day 7	Day 22
Cells counted	2052	3463	2688	2621	3825	3986
Minimum	80.1	29.3	81.3	80.7	73.8	14.8
25% percentile	1041.9	533.3	998.3	843.9	482.2	503.8
Median	2319	1126	1838	1653	982.4	1093
75% percentile	3614.9	1832.3	2659.2	2619.3	1565.4	1797.2
Maximum	9410.5	5920.3	6084.9	9064.5	6544.8	6747.5
Mean	2448	1247^****^	1926^****^	1879^####^	1096^****^	1284^****^
Std. deviation	1643	836.2	1146	1322	757.1	982.8
Std. error of mean	36.28	14.21	22.11	25.83	12.24	15.57
Lower 95% CI of mean	2376.5	1218.7	1882.8	1828.3	1071.5	1253.5
Upper 95% CI of mean	2518.7	1274.4	1969.6	1929.6	1119.6	1314.5

The mean adipocyte sizes in the (inguinal) SCAT of young adult and aged hamsters at days 0 (mock), 7, and 22 after SARS-CoV-2 infection were compared in a two-sided Mann–Whitney test. For intergroup differences, the threshold for statistical significance was set to *p* < 0.05. Values with superscript symbols indicate significant differences (comparison of young adults with aged adults (the effect of age): ^####^*p* < 0.0001 and comparison of mock-treated and SARS-CoV-2-infected groups (the effect of infection): ****p < 0.0001).

**Table 2:** Adipocyte size frequency distribution in VAT.

	Young adults			Aged adults		
	mock	Day 7	Day 22	Mock	Day 7	Day 22
Cells counted	1531	2661	2088	1412	2872	2072
Minimum	68.7	48.2	45.6	72.6	80.1	78.7
25% percentile	922.9	504	651.7	890.7	407.4	457.4
Median	2151	1171	1632	2307	893.3	1226
75% percentile	3769.5	2066.4	2660.2	3894	1538.9	2276.4
Maximum	10854.1	6851.4	8137.5	14228.5	11990.5	19922.5
Mean	2459	1388^****^	1789^****^	2628^#^	1142^****^	1551^****^
Std. deviation	1775	1068	1294	2067	1068	1408
Std. error of mean	45.35	20.7	28.3	55	19.9	30.9
Lower 95% CI of mean	2369.6	1346.9	1733.6	2520.6	1102.4	1490
Upper 95% CI of mean	2547.6	1428	1844.6	2736.4	1180.6	1611.3

The mean adipocyte sizes in the (epidydimal) VAT of young adult and aged hamsters at days 0 (mock), 7, and 22 after SARS-CoV-2 infection were compared in a two-sided Mann–Whitney test. For intergroup differences, the threshold for statistical significance was set to *p* < 0.05 Values with superscript symbols indicate significant differences (comparison of young adults with aged adults (the effect of age): ^#^*p* < 0.05), and comparison of mock-treated and SARS-CoV-2-infected groups (the effect of infection) ****p < 0.0001).

**Fig. 3:**
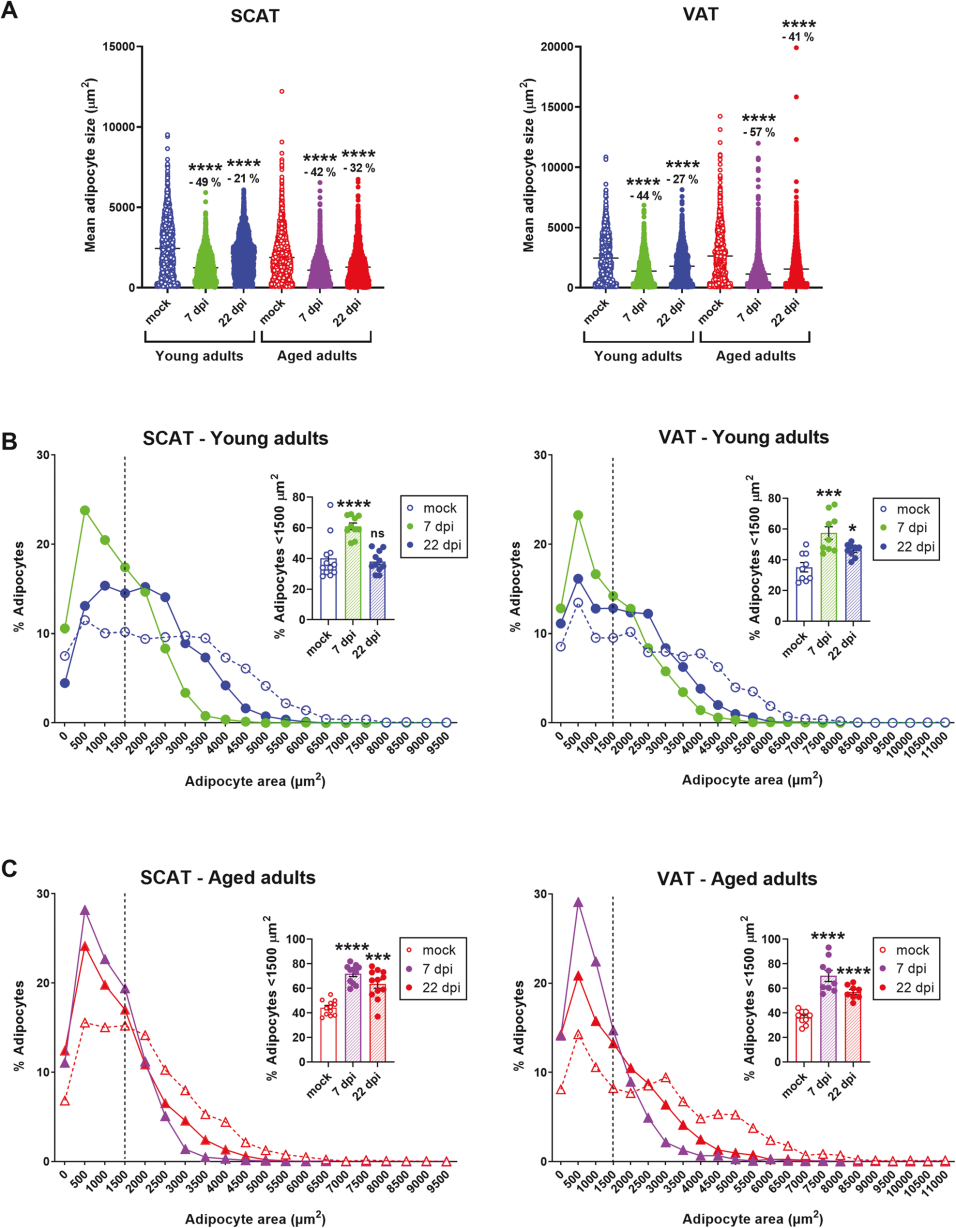
SARS-CoV-2 infection induces a persistent decrease in adipocyte size in SCAT and VAT depots in young adult and aged golden hamsters. Adipocyte size and distribution in the (inguinal) SCAT and (epididymal) VAT were determined by quantitative histomorphometry, at 0 (mock), 7, and 22 dpi in both age groups. **A** Superplots showing the size of individual adipocytes, as well as the mean values (μm^2^), in the SCAT (left) and the VAT (right) of young adult and aged hamsters at 0 (mock), 7, and 22 dpi. Percentage differences in adipocyte size are indicated. **B** Adipocyte size distribution (%) in the SCAT (left) and VAT (right) of young-adult hamsters. The relative frequency of adipocytes <1500 μm^2^ is shown in the insert (mean ± SEM, and individual replicates are shown). **C** Adipocyte size distribution (%) in the SCAT (left) and VAT (right) of aged hamsters. The relative frequency of adipocytes <1500 μm^2^ is shown in the insert (mean ± SEM, and individual replicates are shown). **A**–**C** *n* = 3 animals per group, and a mean (range) of 1035 (649–1500) adipocytes per tissue sample were analyzed. Groups were compared in a two-sided Mann–Whitney test; ^#^ indicates the *P* values for the comparison of young adults and aged adults (the effect of age: ^#^*P* < 0.05, ^##^*P* < 0.01 and ^###^*P* < 0.001) and * indicates the *P* values for the comparison of mock-treated and SARS-CoV-2-infected groups (the effect of infection: *P < 0.05 and **P < 0.01). For intergroup differences, the threshold for statistical significance was set to *P* < 0.05.

Importantly, SARS-CoV-2 infection induced a marked decrease in mean adipocyte size in the SCAT and VAT of both young adult and aged hamsters; this effect was especially maintained at 22 dpi in the latter group (Tables 1 and 2 and Fig. 3A). In SCAT, the decrease in adipocyte size induced by SARS-CoV-2 infection at 7 dpi was similar in young adult and aged hamsters (a 49% decrease vs. mock for young adults, *P* < 0.0001, and a 42% decrease for aged adults, *P* < 0.0001). Importantly, the infection’s impact on adipocyte size in SCAT was still observable at 22 dpi in both age groups but was even more pronounced in the elderly (a 21% decrease in adipocyte size vs. mock for young adults, *P* < 0.0001, and a 32% decrease for aged adults, *P* < 0.0001). Likewise, SARS-CoV-2 infection was associated with a decrease in VAT adipocyte size in young adult and aged hamsters at 7 dpi (a 44% decrease vs. mock for young adults, *P* < 0.0001, and a 57% decrease for aged adults, *P* < 0.0001). This decrease persisted at 22 dpi in both age groups but was greater in aged animals (a 27% decrease vs. mock for young adults, *P* < 0.0001, and 41% decrease for aged adults, *P* < 0.0001). The decrease in SCAT and VAT adipocyte size induced by SARS-CoV-2 infection observed in both age groups suggests that the virus impaired the adipocytes’ lipid storage in fat depots. Thus, we compared the expression levels of lipid-metabolism-related genes in the SCAT and VAT from young adult and aged hamsters at 7 and 22 dpi (Supplementary Fig. S4). At 7 dpi, *Fasn* and *Scd1* expression in the SCAT and VAT of SARS-CoV-2-infected young adult hamsters was significantly lower than in mock-treated controls, as reported by Zickler et al. [37].

Next, we compared the adipocyte size distributions in the SCAT and VAT of mock-treated vs. SARS-CoV-2-infected young adult and aged hamsters (Fig. 3B, C). At 7 dpi, we observed a noticeable leftward and upward shift in the size distribution curve in SARS-CoV-2-infected hamsters, relative to mock-treated animals; hence, the proportion of small (<1500 μm^2^) adipocytes in SCAT and VAT was significantly (~twofold) higher in both SARS-CoV-2-infected age groups. At 22 dpi, the frequency distributions of SCAT and VAT adipocytes had not returned to the mock-treated levels in young-adult animals or (especially) in aged hamsters. Importantly, the SARS-CoV-2-infection-associated increase in the frequency of small (<1500 μm^2^) adipocytes persisted in both the SCAT and VAT of aged hamsters.

Taken as a whole, our results show that the impact of aging per se on WAT cellularity in golden hamsters is depot-specific because lipid metabolism, inflammation, and adipocyte size were altered in the SCAT but not in the VAT. In contrast, the impact of SARS-CoV-2 infection on WAT cellularity was not depot-specific because a reduction in mean adipocyte size (partly due to an alteration in lipid metabolism) was observed in both SCAT and VAT. Importantly, SARS-CoV-2 infection affected WAT cellularity regardless of age but had longer-lasting effects in aged animals.

### SARS-CoV-2 infection is associated with adipocyte-death-related CLSs in the SCAT (but not the VAT) that persist in aged golden hamsters only

Histological assessment of the animals’ WAT revealed tissue architecture differences between SCAT and VAT depots with regard to their response to SARS-CoV-2 infection.

**Fig. 4:**
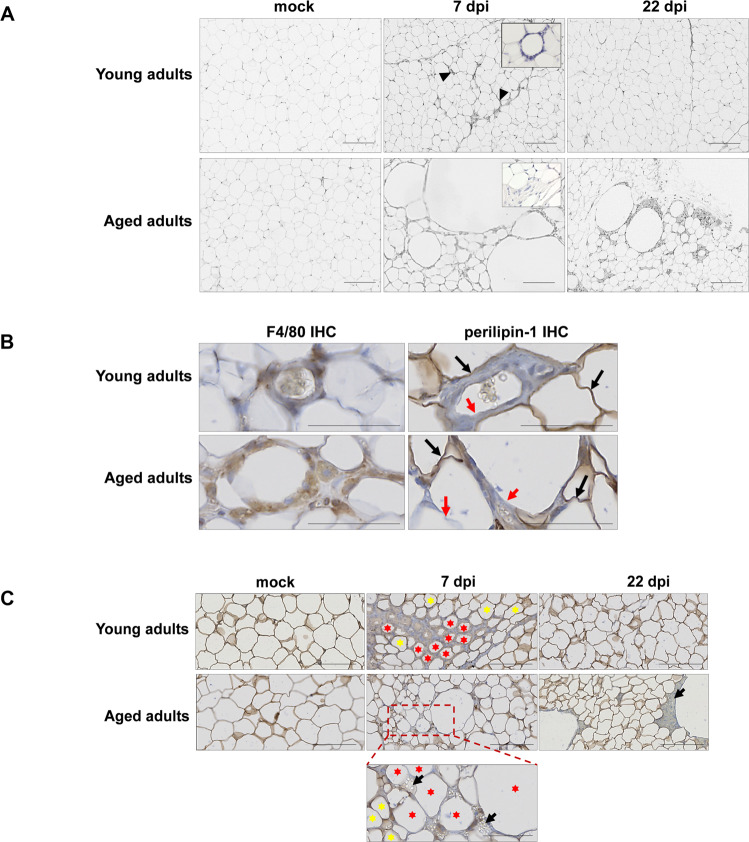
SARS-CoV-2 infection induces adipocyte death in the SCAT, and dead adipocytes are not cleared in aged golden hamsters. **A** Representative microscopy images of the (inguinal) SCAT of young adult and aged hamsters at 0 (mock), 7, and 22 dpi (H&E staining). At 7 dpi, one can note the presence of CLSs in the SCAT of young-adult animals, and the presence of fibrosis in the SCAT of the aged animals (inserts: higher magnification). **B** Representative immunochemical (IHC) staining for F4/80 (left panels) highlighted the presence of macrophages (in brown) clustered around adipocytes. Representative IHC staining for lipid-droplet-specific perilipin-1 (right panels) highlighted live (stained) adipocytes (in brown, black arrows) and damaged/dead (non-stained, red arrows) adipocytes in the SCAT of young adults and aged adults at 7 dpi. **C** Representative images of dead adipocytes in the SCAT of young adult and aged hamsters at 0 (mock), 7, and 22 dpi. IHC staining for perilipin-1 shows live (stained) adipocytes. A higher magnification image is shown for aged adult animals at 7 dpi. Live adipocytes are indicated by yellow stars, and damaged/dead adipocytes are indicated by red stars. Lipid spillovers are indicated by black arrows. **A**–**C** scale bars = 100 μm. **B**, **C** Slides were counterstained with Mayer’s hematoxylin.

In striking contrast to the results for VAT (Supplementary Fig. S5A), SCAT displayed marked microscopic changes upon SARS-CoV-2 infection that were more intense and lasted longer in aged hamsters than in young-adult hamsters (Fig. 4A). At 7 dpi, cellular infiltration into the SCAT was observed in both young adult and aged hamsters. Most of the infiltrating cells were clustered around certain adipocytes. These ring-like cell clusters were reminiscent of the CLSs originally identified in the WAT of mice and humans with obesity [49]. A CLS corresponds to a single damaged or dead adipocyte encircled by lipid-resorbing macrophages [49, 50]. By immunostaining for the macrophage marker F4/80 and for perilipin-1 (an essential lipid-droplet structural protein [51] that is widely used as a marker of adipocyte viability [52–54]), we confirmed that the CLSs observed in the SCAT of SARS-CoV-2-infected hamsters indeed corresponded to macrophages surrounding damaged or dead adipocytes (Fig. 4B). Remarkably, the CLSs that were observed in the SCAT of aged adults had the general characteristics of CLS (i.e., roundish, perilipin-negative central lipid droplet) but were approximately tenfold larger than conventional CLSs. At 22 dpi, while cell infiltrates and CLSs were scarcely observable in the SCAT of young-adult hamsters, they were still present in the SCAT of aged animals (Fig. 4A).

Given that CLSs are surrogate markers of adipocyte death, we next used perilipin-1 staining to visualize dead adipocytes in SCAT samples (Fig. 4C). Surprisingly, at 7 dpi, perilipin-1-negative, dead adipocytes were not randomly distributed in the tissue; they were in close proximity to each other, in both young-adult and aged animals. Furthermore, lipid-rich material was occasionally observed near dead adipocytes in the SCAT of aged animals. At 22 dpi, many perilipin-1-null, large CLSs (surrounded by lipid-rich material) were still present in the SCAT of aged hamsters but not in the SCAT of their younger counterparts. Importantly, the fact that no perilipin-1-negative adipocytes were detected in the VAT at 7 or 22 dpi (Supplementary Fig. S5B) suggested that SARS-CoV-2 infection did not kill adipocytes in this depot.

**Fig. 5:**
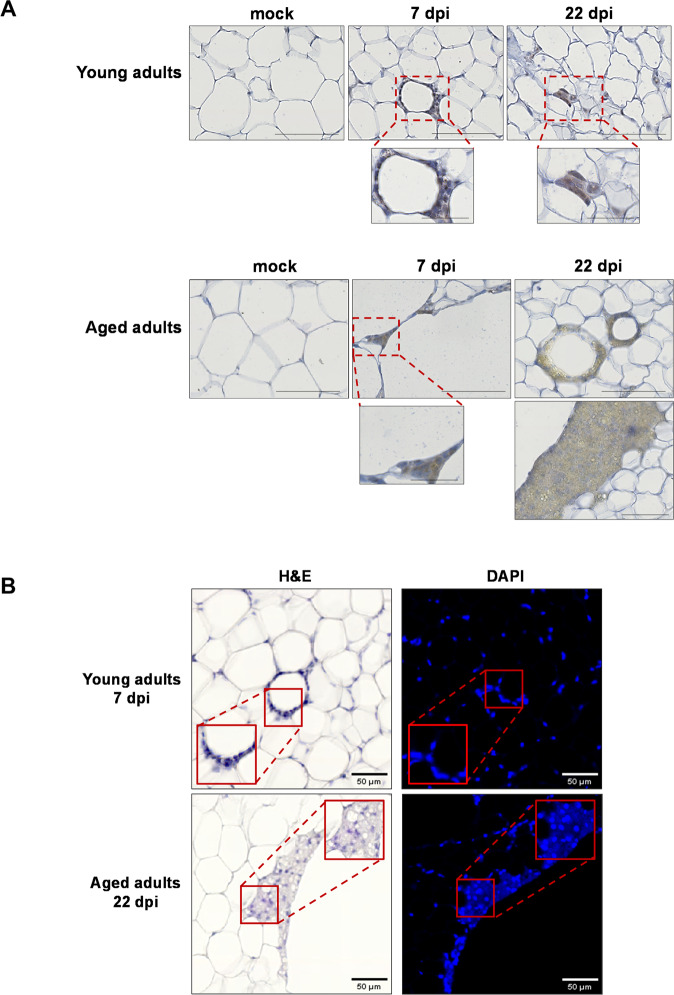
In the SCAT of golden hamsters, the macrophages around dead adipocytes are infected with SARS-CoV-2 and formed syncytia. **A** Representative IHC staining for SARS-CoV-2 spike protein (brown precipitate) in the (inguinal) SCAT of young-adult and aged hamsters at 0 (mock), 7 and 22 dpi (scale bars = 100 μm). Higher magnification images of CLSs are shown (scale bars = 50 μm). Slides were counterstained with Mayer’s hematoxylin. **B** The same CLSs found in the (inguinal) SCAT of young-adult and aged hamsters at, respectively, 7 and 22 dpi, were H&E-stained (left panels) or DAPI-stained (right panels) (scale bars = 50 μm). In left panels, the blue fluorescence indicates DAPI staining for cell nuclei, and higher magnifications are shown that revealed the presence of multinucleated cells.

In adipose tissue, the main target cells for SARS-CoV-2 are mature lipid-laden adipocytes and macrophages [21, 37, 42]. Furthermore, SARS-CoV-2-infection-induced adipocyte death has been reported [42]. Hence, we used immunohistochemistry staining to screen SCAT samples for the SARS-CoV-2 spike protein (Fig. 5A). In the SCAT of young-adult hamsters, a significant staining was found in macrophages clustered around adipocytes (7 dpi) or in macrophages close to adipocytes (22 dpi); hence, SARS-CoV-2 spike antigen had accumulated in these macrophages. In the SCAT of 7-dpi-infected aged animals, intense staining was observed in the macrophages clustered around conventional CLSs (Supplementary Fig. S5C) and large CLSs. Interestingly, this staining was more diffuse (indicating the presence of smaller amounts of spike protein) at 22 dpi than at 7 dpi.

Syncytia formation is a hallmark of SARS-CoV-2 infection that results from the activation of the spike protein at the plasma membrane of infected cells [55–59]. Moreover, in the WAT of mice and humans with obesity, macrophages that are organized as CLSs fuse to form syncytia that sequester and scavenge the residual adipocyte lipid droplet and ultimately form multinucleated giant cells [49, 60]. Thus, we assessed whether SARS-CoV-2-infected macrophages that cluster around dead adipocytes in the SCAT of infected young-adult and aged hamsters may have formed syncytia. Fluorescent labeling of nuclei indeed revealed the presence of some multinucleated cells at the levels of CLSs; indicative of syncytia formation during SARS-CoV-2 infection of the SCAT (Fig. 5B).

In summary, these results highlight the depot-specific consequences of SARS-CoV-2 infection of WAT. Indeed, CLSs (dead adipocytes surrounded by infected macrophages—some of them forming syncytial multinucleated cells) were observed in the SCAT but not in the VAT. Importantly, the presence and persistence of conventional CLSs but also abnormally large CLSs in the SCAT of aged hamsters might indicate an age-related defect in the clearance of dead adipocytes by infected macrophages following SARS-CoV-2 infection.

### Plasma lipid signatures differentiate SARS-CoV-2-infected young-adult golden hamsters from SARS-CoV-2-infected aged golden hamsters

**Fig. 6:**
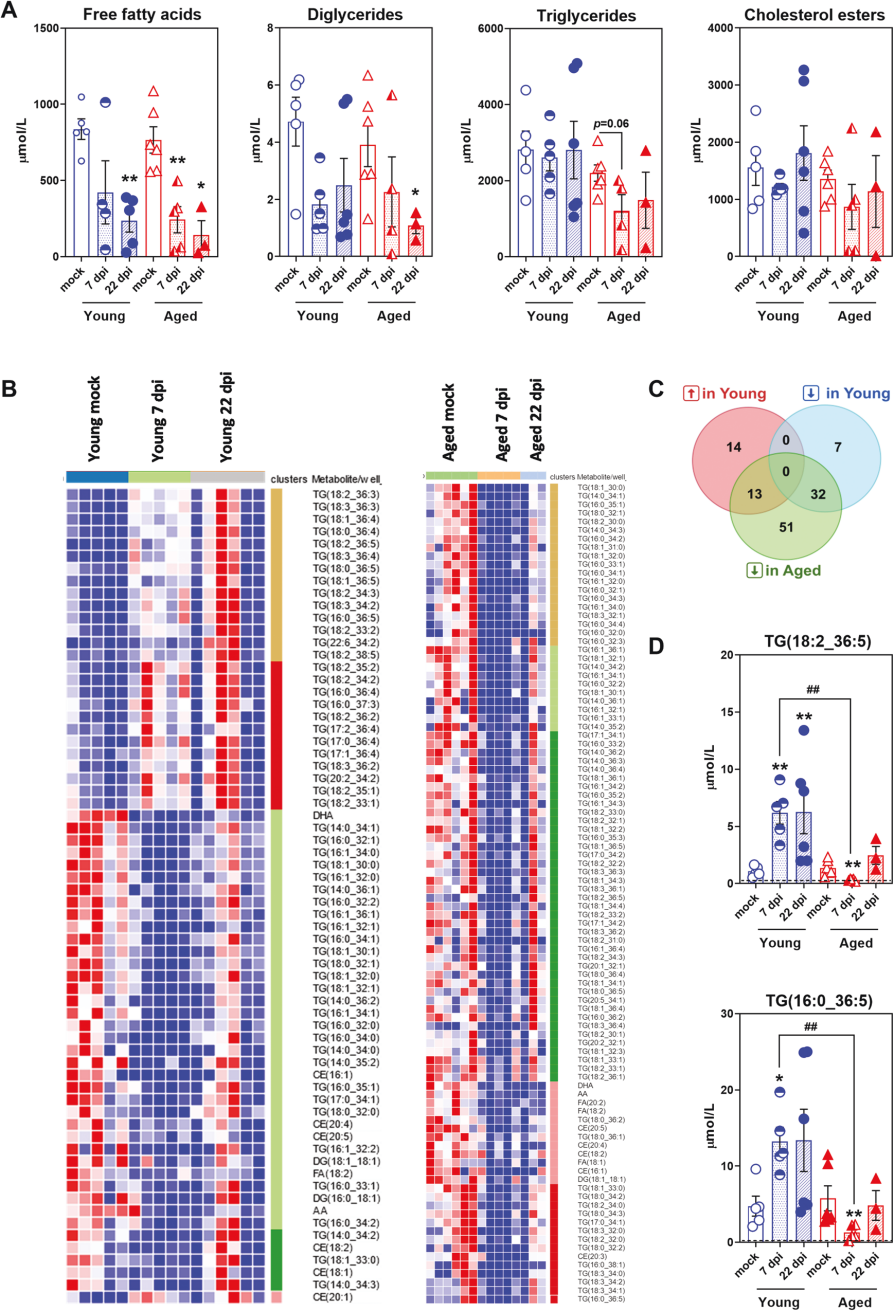
SARS-CoV-2 infection differently disturbs plasma lipid metabolism in young-adult and aged golden hamsters. Plasma levels of free fatty acids (FFAs), diglycerides (DGs), cholesterol esters (CEs) and triglycerides (TGs) were quantified by ultrahigh-pressure liquid chromatography-tandem mass spectrometry (UPLC-MS/MS) in young-adult and aged hamsters at 0 (mock), 7 and 22 dpi. **A** Plasma levels of FFAs (*n* = 5 molecules), DGs (*n* = 4 molecules), TGs (*n* = 154 molecules) and CEs (*n* = 16 molecules) at days 0 (mock), 7 and 22 post-SARS-CoV-2 infection. Data are expressed as mean ± SEM μmol/L, and individual replicates are shown. **B** Heatmaps of significantly changed lipid species (fold change >1.5, *P* < 0.05) at 7 dpi in young-adult hamsters and aged hamsters (Young adults: *n* = 66 lipid species (27 increased, 39 decreased), and aged adults: *n* = 96 lipid species (all decreased)). Differential intensities in red and blue colors denote increased or reduced levels, respectively (less concentrated: dark blue, most concentrated: dark red). **C** Venn diagrams (https://bioinformatics.psb.ugent.be/) showing the number of distinct or overlapping lipid species that are increased (↑) or decreased (↓) upon SARS-CoV-2 infection (7 dpi) in young-adult and aged hamsters. **D** Plasma levels of TG(18:2_36:5) and TG(16:0_36:5) in young-adult and aged hamsters at days 0 (mock), 7 and 22 post-SARS-CoV-2 infection. Data are expressed as mean ± SEM mmol/L, and individual replicates are shown. Dotted lines indicate the limit of detection (LOD) of the assay for each lipid species. **A**–**D** *n* = 5 young adults mock, *n* = 6 aged adults mock, *n* = 5 young adults 7 dpi, *n* = 5 aged adults 7 dpi, *n* = 6 young adults 22 dpi, and *n* = 3 aged adults 22 dpi. Young = young-adult hamsters, Aged = aged adult hamsters. **A**, **D** Groups were compared in a two-sided Mann–Whitney test; # indicates the *P* values for the comparison of young adults and aged adults (the effect of age: ^##^*P* < 0.01) and * indicates the *P* values for the comparison of mock-treated and SARS-CoV-2-infected groups (the effect of infection: *P < 0.05 and **P < 0.01). For intergroup differences, the threshold for statistical significance was set to *P* < 0.05.

To assess whether the SARS-CoV-2-infection-induced WAT alterations were associated with changes in blood lipid profiles, a targeted metabolomic analysis was performed on plasma samples of young-adult and aged hamsters at 0 (mock), 7, and 22 dpi (Fig. 6). Data corresponding to four lipid classes i.e., free fatty acids (FFAs) (*n* = 5 molecules), diglycerides (DGs) (*n* = 4 molecules), triglycerides (TGs) (*n* = 154 molecules), and cholesterol esters (CEs) (*n* = 16 molecules) were specifically analyzed. As shown in Fig. 6A, SARS-CoV-2 infection induced a statistically significant decrease in FFAs and DGs plasma concentrations, especially in aged hamsters, whereas no significant changes were observed for TGs and CEs in either age group and whatever the time post infection (with the exception of TG levels that tended to decrease in aged hamsters at 7 dpi (*P* < 0.06)). However, out of the 179 lipid species that were quantified, 66 in young adult vs. 96 in aged hamsters showed significantly altered levels at 7 dpi in comparison to the mock respective groups (fold change >1.5, *P* < 0.05); this indicates that SARS-CoV-2 infection perturbs lipid metabolism more importantly in aged than young-adult hamsters. To obtain an overview of the main changes in our metabolomic data, we next performed a heatmap and clustering analysis using these lipid species (Fig. 6B). In young adults, 27 lipid species (out of 66) were increased and 39 lipid species (out of 66) were decreased upon SARS-CoV-2 infection, while in aged animals all of the 96 lipid species were decreased upon SARS-CoV-2 infection (Supplementary Table S2 and Fig. 6C). Interestingly, in young adults the triglyceride species that were increased were enriched in polyunsaturated fatty acids (PUFAs) and the ones that were decreased were enriched in saturated fatty acids (SFAs) and monosaturated fatty acids (MUFAs) (Supplementary Table S2), as reported by Zickler and colleagues [37]. Venn diagrams were then used to display a comparison of the lists of increased or decreased lipid species in young-adult and aged hamsters (Fig. 6C and Table 3). Interestingly, 13 lipid species were increased upon SARS-CoV-2 infection in young adult but decreased in aged hamsters, such as TG(18:2_36:5) and TG(16:0_36:5) (Fig. 6D), 32 lipid species were decreased upon SARS-CoV-2 infection in both age groups, and 51 lipid species were specifically decreased in aged hamsters only (Fig. 6C and Table 3).

**Table 3:** Shared and specific plasma lipids that are modulated by SARS-CoV-2-infection in young-adult and aged golden hamsters.

	#	Lipids
↑ Young adults ↓ Aged adults	13	TG(18 :3_36 :2), TG(18 :1_36 :4), TG(18 :2_33 :2), TG(18 :0_36 :4) TG(18 :1_36 :5), TG(18 :2_36 :5), TG(16 :0_36 :5), TG(18 :3_36 :3) TG(18 :2_34 :3), TG(18 :3_34 :2), TG(18 :2_33 :1), TG(18 :3_36 :4) TG(18 :0_36 :5)
↓ Young adults ↓ Aged adults	32	TG(16 :0_33 :1), TG(16 :0_34 :2), TG(16 :1_34 :1), TG(14 :0_34 :3) TG(17 :0_34 :1), TG(16 :1_36 :1), TG(16 :0_32 :2), AA, CE(18 :2) DG(18 :1_18 :1), CE(20 :5), CE(16 :1), TG(18 :1_32 :0), FA(18 :2) TG(16 :0_34 :1), TG(18 :0_32 :1), TG(14 :0_35 :2), TG(18 :1_32 :1) TG(18 :1_30 :0), TG(18 :1_30 :1), TG(14 :0_34 :1), TG(14 :0_34 :2) TG(18 :1_33 :0), TG(16 :1_34 :0), CE(20 :4), TG(14 :0_36 :1), DHA TG(16 :1_32 :1), TG(16 :1_32 :0), TG(16 :0_32 :0), TG(16 :0_35 :1) TG(16 :0_32 :1)
↑ Young adults	14	TG(17 :2_36 :4), TG(17 :1_36 :4), TG(18 :2_36 :2), TG(20 :2_34 :2) TG(18 :2_36 :3), TG(16 :0_36 :4), TG(18 :2_35 :1), CE(20 :1) TG(18 :2_34 :2), TG(22 :6_34 :2), TG(18 :2_35 :2), TG(18 :2_38 :5) TG(17 :0_36 :4), TG(16 :0_37 :3)
↓ Young adults	7	CE(18 :1), DG(14 :0_36 :2), TG(18 :0_32 :0), TG(14 :0_34 :0) TG(16 :0_34 :0), DG(16 :0_18 :1), TG(16 :1_32 :2)
↓ Aged adults	51	TG(20 :5_34 :1), TG(18 :1_34 :3), TG(18 :3_34 :0), TG(18 :3_32 :0) TG(16 :0_35 :2), TG(18 :2_36 :1), TG(16 :1_34 :3), TG(18 :2_31 :0) TG(18 :1_32 :2), TG(14 :0_36 :2), TG(18 :1_33 :1), TG(16 :0_35 :3) TG(17 :0_34 :2), TG(18 :0_34 :2), TG(18 :2_30 :0), TG(18 :1_36 :1) TG(18 :1_34 :1), TG(16 :1_34 :2), TG(14 :0_36 :3), TG(18 :0_36 :2) FA(20 :2), TG(20 :1_32 :1), TG(18 :0_34 :3), TG(18 :3_36 :1) TG(14 :0_36 :4), TG(16 :0_32 :3), CE(20 :3), TG(16 :0_34 :4) TG(18 :1_31 :0), TG(18 :0_32 :2), TG(16 :1_33 :1), TG(16 :1_36 :4) TG(16 :0_34 :3), TG(17 :1_34 :1), FA(18 :1), TG(20 :2_32 :1) TG(18 :2_30 :1), TG(18 :3_32 :1), TG(18 :2_32 :2), TG(18 :2_33 :0) TG(17 :1_34 :2), TG(18 :1_34 :4), TG(16 :0_33 :2), TG(18 :2_32 :1) TG(18 :1_32 :3), TG(18 :2_32 :0), TG(18 :0_36 :1), TG(18 :2_34 :0) TG(18 :3_34 :1), TG(16 :0_38 :1), TG(16 :0_36 :2)

At 7 dpi, lipids significantly increased upon infection in young-adult but decreased in aged hamsters (*n* = 13), lipids significantly decreased upon infection in both age groups (*n* = 32), lipids significantly increased upon infection in young-adult hamsters only (*n* = 14), lipids significantly decreased upon infection in young-adult hamsters only (*n* = 7), and lipids significantly decreased upon infection in aged hamsters only (*n* = 51). Fold change >0.5, *P* < 0.05.

These results show that SARS-CoV-2-infection-induced alterations of plasma lipid profiles significantly differ between young-adult and aged hamsters; more changes were evidenced in the elderly. Considering the role of WAT in the control of systemic lipid metabolism, we hypothesize that the more severe impact of SARS-CoV-2 infection observed in the WAT of aged animals contributes, at least partly, to this lipid profile phenotype.

## Discussion

Like obesity, advanced age is a major risk factor for clinically severe COVID-19. Indeed, mortality rates are significantly higher in adults with obesity and in elderly adults [11–17, 61–65]. Interestingly, obesity and aging are both associated with the pathological expansion of WAT in general and VAT depot in particular; this suggests that WAT remodeling has a role in the obesity- and aging-associated increase in COVID-19 severity. The recent discovery that WAT is a SARS-CoV-2 reservoir [38, 39, 66–69] emphasizes the need to characterize the role of this tissue in the pathogenesis of COVID-19 in these at-risk populations. While most research has focused on patients with obesity, little is known about SARS-CoV-2-infection-related WAT’s pathophysiology in aged individuals. Hence, in the golden hamster model, we sought to explore age-related differences in the response of inguinal SCAT and epididymal VAT depots to SARS-CoV-2 infection.

We confirmed that relative to their younger counterparts, aged hamsters developed more severe (and sometime lethal) disease manifestations [16, 17]. Moreover, we showed that SARS-CoV-2 infection induces profound WAT remodeling, albeit in different ways in young-adult vs. aged hamsters.

SARS-CoV-2 infection led to a decrease in the mean adipocyte size in both SCAT and VAT. This decrease was associated with reduced expression of lipid-synthesis-related genes (such as *Fasn* and *Scd1*) and thus suggested that SARS-CoV-2 infection impairs triglyceride storage capacity in the SCAT and the VAT. These findings are in line with previous reports on the impact of SARS-CoV-2 infection on both local (WAT) and systemic lipid metabolism in hamsters and humans [37, 70, 71]. The non-depot-specific effects of SARS-CoV-2 infection on WAT were observed soon after infection (7 dpi) and lasted up to 22 dpi in both young-adult and aged hamsters. However, these alterations were greater in aged animals and so might participate to the metabolic dysregulations associated with severe COVID-19 [72].

We showed for the first time that SARS-CoV-2-infected animals also displayed WAT-depot-specific alterations upon infection, i.e., the presence of CLSs in the SCAT but not in the VAT. CLSs were originally described in the WAT of individuals with obesity and can be viewed as markers of adipocyte death and local inflammation [49, 73]. Our results demonstrate that in golden hamsters, a SARS-CoV-2 infection kills adipocytes in the SCAT but not in the VAT. Since (i) SARS-CoV-2 can disseminate to WAT [37] and (ii) viral infection of mature adipocytes reduces cell viability [42], we speculate that the CLSs observed in the SCAT of SARS-CoV-2-infected animals are histological hallmarks of virus-induced adipocyte death. It is also noteworthy that the dead adipocytes were clumped together in some areas and were not distributed randomly through the tissue. This suggests that SARS-CoV-2 might spread through cell-to-cell transmission in the SCAT—a mechanism that has been observed in vitro [74].

WAT macrophages are reportedly permissive to SARS-CoV-2 infection [21]. We found that the macrophages around dead adipocytes expressed the SARS-CoV-2 spike protein, which is indicative of direct infection or indirect infection (i.e., via adipocyte-to-macrophage transmission [74]) of these cells. Interestingly, some of these macrophages formed syncytial multinucleated cells; this suggests the proteolytic activation of the SARS-CoV-2 spike protein at their plasma membrane [75]. It could be envisioned that SARS-CoV-2-induced macrophage fusion will also facilitate the transfer of the viral genome to neighboring cells, as it has been shown for other viruses such as human immunodeficiency virus, respiratory syncytial virus, and herpes simplex virus [76]. While severe cases of COVID-19 are associated with extensive lung damage and the presence of infected multinucleated syncytial pneumocytes and leukocytes [58, 77], it remains to be determined whether syncytia formation by SARS-CoV-2-infected macrophages is more frequent in the SCAT of aged hamsters. Thus, the crown-like pattern of infected macrophages around dead adipocytes (probably killed by the viral infection) might constitute a micro-anatomical sign of SARS-CoV-2 infection in the SCAT. Remarkably, CLSs were much more frequent in the SCAT of aged hamsters than in the SCAT of younger animals, likely due to age-related inflammation in this fat depot. Hence, age might increase SCAT’s susceptibility to SARS-CoV-2 infection and widespread cell death. Other researchers have found that a high CLS count in WAT is correlated with worse clinical outcomes in patients with obesity [50, 78]. Similarly, the CLS count in breast and periprostatic WAT has been linked to poor clinical outcomes in patients with breast or prostate cancer, respectively [79, 80]. Recently, three-dimensional (3D) imaging has been used to accurately assess CLS sizes and counts, and to classify CLSs by their shape and by the number and type of macrophages surrounding damaged adipocytes [81]; this will allow to highlight (potential) correlation between CLS features in the SCAT of SARS-CoV-2-infected individuals and clinical conditions and/or progression to COVID-19 severity in at-risk populations, such as older adults.

In addition to high numbers of conventional CLSs, we also observed abnormally large CLSs in the SCAT of aged hamsters (relative to younger counterparts) at 7 dpi and, especially, at 22 dpi. These observations indicate an inappropriate restorative/repair response to the adipocyte death caused by SARS-CoV-2 infection in the SCAT of aged animals. These surprisingly large CLSs are reminiscent of (i) the cyst-like structures observed in the SCAT of individuals with both obesity and diabetes [82], and (ii) the oil (lipid) cysts occurring in the necrotic fatty breast tissue [83]. Adipocytes contain a large central lipid droplet; the clearance of dead adipocytes by macrophages requires efficient scavenging of the lipids that spill out of dead or damaged cells. Aging reduces the ability of macrophages (including WAT macrophages) to clear dead cells (a process named efferocytosis) [84–86]. We hypothesize that the large, oil-cyst-like structures seen in the SCAT of SARS-CoV-2-infected, aged hamsters up to 22 dpi result from the inefficient scavenging and elimination of dead adipocytes by surrounding macrophages. Interestingly, it was recently reported that lung macrophages from severe COVID-19 patients have compromised efferocytic capacity [87]. Moreover, aging is known to be associated with a reduction in both the number of adipocyte precursors and the latter’s ability to differentiate into mature, lipid-laden adipocytes [88, 89]. Thus, the accumulation of tissue damage in the SCAT of aged hamsters may result from both aged-related inefficient efferocytotic function of tissue’s macrophages and age-related defective adipocyte turnover and de novo adipogenesis for the replacement of dead adipocytes.

Furthermore, we found that the SARS-CoV-2-infection-induced profound WAT remodeling was associated with systemic alterations; infection led to marked changes in plasma lipid profiles in the two age groups. Importantly, differential plasma lipid signature discerned between young-adult and aged hamsters; more changes were evidenced in the elderly, some of which being only observed in this age group. Whether the unique plasma lipid signature of SARS-CoV-2-infected aged hamsters align with infection-induced WAT changes and with disease severity remains to be demonstrated. However, alterations of the plasma lipid profile are frequently observed in patients with COVID-19, and accumulating evidence suggests that systemic lipid metabolism may predict disease progression in patients with severe COVID-19 [70, 71, 90–93].

**Fig. 7:**
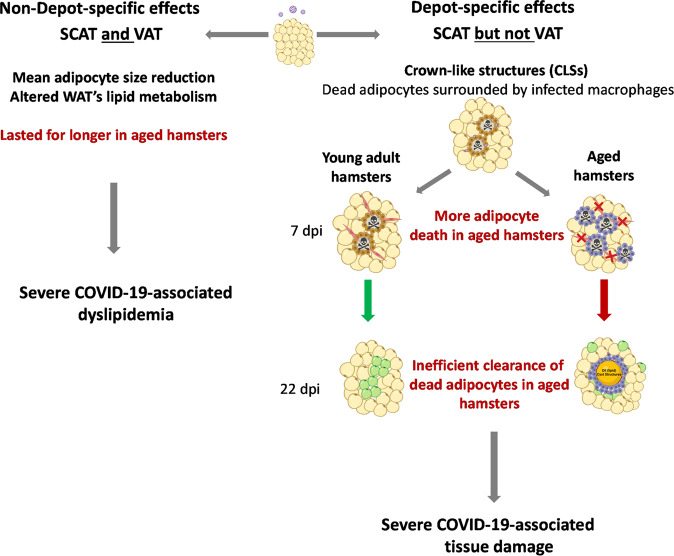
Age-related differences in WAT’s response to SARS-CoV-2 infection in golden hamsters. Schematic diagram showing the non-depot-specific (left) and the depot-specific (right) effects of a SARS-CoV-2 infection on WAT in young-adult vs. aged golden hamsters. The viral infection perturbed lipid metabolism in both the (inguinal) SCAT and the (epididymal) VAT but induced CLS formation—indicative of infection-induced adipocyte death—in SCAT only. Importantly, massive adipose death was evident in the SCAT of aged hamsters. The repair phase post infection-induced injury was effective in young adults but not in aged animals. We hypothesize that the accumulation of tissue damage in the SCAT of aged animals results from both the defective clearance of dead adipocytes by surrounding macrophages having age-related compromised efferocytic capacity, and/or the absence of replacement of dead adipocytes by newly formed adipocytes due to age-related impaired adipogenesis. CLSs crown-like structures. Preadipocytes are shown in red, and newly formed adipocytes are shown in green. Both non-depot-specific and depot-specific WAT remodeling upon SARS-CoV-2 infection might participate to COVID-19 severity in aged individuals. Created with BioRender.com.

In conclusion, we described profound WAT remodeling upon SARS-CoV-2 infection that lasted longer in aged golden hamsters than in young-adult golden hamsters (Fig. 7). Some manifestations of WAT remodeling in response to SARS-CoV-2 infection occurred in both SCAT and VAT, i.e., the decreased adipocyte size resulting from infection-induced alteration of tissue’s lipid metabolism. Other manifestations of WAT remodeling in response to SARS-CoV-2 infection were selectively present in SCAT, i.e., the presence of dead adipocytes surrounded by a ring of macrophages. Importantly, aging increased SCAT’s susceptibility to SARS-CoV-2-infection-induced adipocyte death and was associated with an inefficient resolution of infection-induced tissue damage—possibly as a result of the compromised capacity of tissue’s macrophages to clear dead adipocytes. Finally, we showed that SARS-CoV-2-infection-induced WAT remodeling was associated with age-related changes in plasma lipid profiles. Thus, our findings may provide mechanistic insights for the accumulation of tissue (including WAT) damage associated with severe COVID-19, and may open up new perspectives regarding the involvement of WAT in SARS-CoV-2 infections in at-risk populations, such as older individuals.

## Materials and methods

### Biosafety and ethics

All work using live SARS-CoV-2 virus was performed within the biosafety level 3 laboratory (BSL3) of the Institut Pasteur de Lille, and complied with current national and institutional regulations and ethical guidelines (Institut Pasteur de Lille/B59-350009). All experimental protocols were approved by the institutional regional ethical committee “Comité d’Ethique en Experimentation Animale” (CEEA) 75. The study was authorized by the “Education, Research and Innovation Ministry” under registration number APAFIS#25041-2,020,040,917,227,851 v3. Animals were group-housed in HEPA-filtered cage systems enriched with nesting materials.

### Animals, virus, and infection

Male golden (Syrian) hamsters (*Mesocricetus auratus*), 2-months-of-age (the “young adults” group) and 22-months-of-age (the “aged adults” group), were purchased from Janvier Labs (Le Genest-Saint-Isle, France). Hamsters were fed a standard rodent chow (SAFE® A04, Augy, France) and were given water ad libitium. The hCoV-19_IPL_France strain of SARS-CoV-2 (NCBI MW575140) was isolated on TMPRSS2 expressing Vero-81 cells and passaged 4 times on these cells before usage. For infection, hamsters were anesthetized by intraperitoneal injection of ketamine (100 mg/kg) (Boehringer-Ingelheim, Lyon, France), atropine (0.75 mg/kg) (Agettant, Lyon, France) and valium (2.5 mg/kg) (Roche, Boulogne-Billancourt, France), and intranasally infected with 100 µl of DMEM containing (or not, for mock-treated control animals) 2 × 10^4^ TCID_50_ (50% Tissue Culture Infectious Dose) of SARS-CoV-2.

### Antibodies

For the detection and localization of SARS-CoV-2, rabbit anti-SARS-CoV-2-spike glycoprotein primary antibody (ab272504, Abcam, Cambridge, UK) and HRP-conjugated goat anti-rabbit secondary antibody (Vector Laboratories, Burlingame, CA, USA) were used. For the detection and localization of macrophages, rabbit polyclonal anti-F4/80 (ab100790, Vector Laboratories) and HRP-conjugated goat anti-rabbit secondary antibody (Vector Laboratories) were used. For the detection of lipid droplets, rabbit polyclonal anti-perilipin-1 primary antibody (ab3526, Abcam) and HRP-conjugated goat anti-rabbit secondary antibody (Vector Laboratories) were used.

### Sample collection

During the experimental period, body weights were recorded daily from day 0 to day 22 post infection (dpi). For tissue collection, animals were euthanized by intraperitoneal injection of phenytoin/pentobarbital sodium (EUTHASOL® VET, DECHRA Veterinary Products, Montigny-le-Bretonneux, France) (140 mg/kg). Lungs and adipose tissues (subcutaneous (inguinal) SCAT, and visceral (epididymal) VAT) were collected from non-infected (mock) hamsters and from SARS-CoV-2-infected hamsters at 7 and 22 dpi. Half of the SCAT and VAT was fixed in 4% paraformaldehyde for 7 days, transferred to 70% ethanol and embedded in paraffin for histology, histomorphometry and immunohistochemistry. The remaining halves SCAT and VAT were stored at −80 °C for further gene expression analyses. Half-lung was used for viral load quantification, and the other half was used for gene expression analyses. Blood samples were collected from mock-infected and SARS-CoV-2-infected hamsters for plasma metabolomic analysis.

The experimental procedure is presented in Supplementary Fig. 1.

### RNA isolation and real-time quantitative PCR

Tissues (lungs, SCAT, and VAT) were homogenized in 1 ml of RA1 buffer (NucleoSpin RNA kit, Macherey-Nagel, Düren, Germany) and 20 mM of Tris(2-carboxyethyl) phosphine hydrochloride (TCEP). Total RNAs in tissue homogenates were extracted using the NucleoSpin RNA kit (Macherey-Nagel).

For quantification of SARS-CoV-2 RNA copy numbers, one-step qPCR assay was performed using 5 µl of RNA and Takyon One-Step Low Rox RT probe Master Mix (Eurogentec, Angers, France) with specific primers and probe targeting the *E* gene that encodes the viral envelope (E) protein (probe sequence: FAM-ACACTAGCCATC-CTTACTGCGCTTCG-MGB), following manufacturer’s instructions. A synthetic gene containing the SARS-CoV-2 E gene (position 26269-26381) (Genewiz, Paris, France) was used to construct the standard curve.

For quantification of the expression levels of the other genes, cDNA was generated using the High-Capacity cDNA Archive Kit (ThermoFisher Scientific, Illkirch-Graffenstaden, France). Quantitative real-time PCR (qPCR) was then performed in triplicates using the Power SYBR^®^ Green PCR Master Mix (ThermoFisher Scientific) and QuantStudio™ 12 K Flex Real-Time PCR Instrument (Applied Biosystems, Foster City, CA, USA). Specific primers were designed using the Primer Express^TM^ v3 software (ThermoFisher Scientific), and ordered from Eurofins Genomics (Ebersberg, Germany). Forward and reverse primer sequences are listed in Supplementary Table S1. Relative expression levels were normalized to the levels of *Actg1* (coding for gamma actin) (lungs) or *GusB* (coding for glucuronidase beta) (WAT). The 2^-ΔΔCt^ method was used to calculate relative expression changes.

### Adipose tissue histology and histomorphometry

Paraformaldehyde-fixed paraffin-embedded SCAT and VAT were sectioned (5 μm-thick), de-paraffinized, rehydrated, and stained with hematoxylin and eosin (H&E) for histological examination. For measurement of adipocyte size by histomorphometry, at least four fields per H&E-stained slide were visualized and images were digitally captured using a digital camera (AxioCam HRc, Zeiss, Göttingen, Germany) connected to an optical microscope (Axioplan 2 Imaging, Zeiss) at ×10 magnification. Adipocyte areas (in μm^2^) were averaged to obtain mean cross-sectional area, using an in-house macro specifically developed for automated image analysis of WAT on the Fiji-ImageJ software (NIH freeware), and were binned by area to calculate the distribution of cell sizes within the sections (3 animals per group, an average of 1035 adipocytes per tissue sample were measured with a range of 649–1500 cells per tissue sample). Adipocytes with a disrupted membrane border were neither traced for analysis nor were those with incomplete borders at the image frame.

### Immunohistochemistry

Paraffin-embedded SCAT and VAT were sectioned (7-μm-thick), dewaxed, and rehydrated through toluene and graded alcohol, respectively, before epitope unmasking by boiling for 5 min in sodium citrate solution (pH 6.0). A 1% solution of H_2_O_2_ in DPBS was used for 20 min to block endogenous peroxidase activity. Slides were then blocked for 3 h at room temperature in blocking solution (PBS containing 5% normal goat serum and 5% BSA) prior to blocking endogenous biotin- or avidin-binding proteins, using the Avidin/Biotin Blocking kit (Vector Laboratories) as recommended by the manufacturer. Samples were then washed three times in PBS and exposed overnight at 4 °C to primary antibodies diluted in 2% blocking solution (spike (1:5000), F4/80 (1:50), perilipin-1 (1:500)), followed by incubation with HRP-conjugated goat anti-rabbit secondary antibody, rinsed, and incubated with VECTASTAIN®Elite ABC-HRP kit (Vector laboratories) following manufacturer’s instructions. Slides were washed three times in PBS and the 3,3’-diaminobenzidine (DAB) chromogen DAB Peroxidase (HRP) Substrate Kit (Vector Laboratories) was added on each slide. Subsequently, sections were counterstained in Mayer’s hematoxylin (Merck, Darmstadt, Germany), and glass coverslips mounted using Dako mounting medium. To visualize syncytia in SCAT samples, fluorescent labeling of nuclei was performed. Sections were stained with 4’,6-diamidino-2-phenylindole (DAPI) (Sigma-Aldrich, St Louis, MO, USA) for 10 min and coverslips were then mounted on slides using a fluorescence mounting medium (Agilent Technologies, Santa Clara, CA, USA). Mounted slides were stored in the dark and at 4 °C until image acquisition. All images were acquired using the Axio Scan.Z1 slide scanner and ZEN (Blue edition) 2012 software (Carl Zeiss, Oberkochen, Germany).

### Plasma lipid profiling by targeted UPLC-MS/MS assay, and data analysis

To compare the lipid signatures in SARS-CoV-2-infected young-adult and aged hamsters, ultrahigh-pressure liquid chromatography-tandem mass spectrometry (UPLC-MS/MS) was used. Ethylenediaminetetraacetic (EDTA) plasma samples were processed using the MxP^®^ Quant 500 kit (BIOCRATES Life Sciences AG, Innsbruck, Austria) according to the manufacturer’s instructions. Briefly, 10 μL of each EDTA plasma sample (*n* = 5 young adults mock, *n* = 6 aged adults mock, *n* = 5 young adults 7 dpi, *n* = 5 aged adults 7 dpi, *n* = 6 young adults 22 dpi, and *n* = 3 aged adults 22 dpi) was loaded onto a filter containing internal standards for normalization. Filters were dried under a stream of nitrogen and incubated with derivatization reagent phenyl-isothiocyanate 5%. Dried analytes were subsequently extracted with 5 mmol/L ammonium acetate in methanol and analyzed by UPLC-MS/MS. The targeted analysis allowed for the identification and quantification of 630 metabolites (including nine different lipid classes) (https://biocrates.com/mxp-quant-500-kit/) detected by MS/MS after UPLC separation and flow injection analysis (FIA) (SCIEX 5500 Triple Quad System, SCIEX, Framingham, MA, USA). Data were recorded using the Analyst software (SCIEX) and transferred to the MetIDQ^TM^ software (BIOCRATES) which was used for further data processing i.e., technical validation, quantification, and data export. For each metabolite, peaks were quantified using area-under-the-curves. The analytical method has been fully validated by the kit’s manufacturer according to FDA and EMA guidelines.

For DGs, both fatty acid identities were obtained, while for TGs only one of the 3 fatty acids was definitively identified. Consequently, TGs are annotated as TG (R1-R2 + R3) where R1 is the first fatty acid and R2 + R3 is the sum of chain lengths and desaturations for fatty acids 2 and 3.

Data corresponding to four classes of lipids i.e., free fatty acids, diglycerides, cholesterol esters, and triglycerides were specifically analyzed (*n* = 320 molecules, raw data available upon request). Only lipids that were above limit of detection (LOD) and limit of quantification (LOQ) in at least three replicates in the young-adult-mock group were kept for further analyses (*n* = 179 molecules). Differential lipids with age and/or infection status were obtained using a false discovery rate (FDR) < 0.05.

Statistical comparisons were calculated using the limma package implemented in Phantasus (Zenkova et al. [94]; https://genome.ifmo.ru/phantasus). Heatmaps and k-means clustering were performed in Phantasus after filtering for differentially present lipids. Statistical significance was established once average lipid concentrations were fold change >1.5 and *P* < 0.05 in group comparisons.

### Statistical analyses

Values are presented as individual replicates and means ± SEM, unless otherwise stated. Statistical analyses were performed using GraphPad Prism v9 software. A Mann–Whitney *U* test was used to compare two groups unless otherwise stated. Comparisons of more than two groups were analyzed with the One-way ANOVA Kruskal–Wallis nonparametric test, followed by the Dunn’s posttest. The symbols * and # represent, respectively, the significant differences between mock-treated and SARS-CoV-2-infected groups (infection effect), and between young-adult and aged adult groups (age effect). *P* < 0.05 was considered statistically significant.

## Supplementary information

Supplementary Table S1

Supplementary Table S2

Legends to Supplementary Figures

Supplementary Figure S1

Supplementary Figure S2

Supplementary Figure S3

Supplementary Figure S4

Supplementary Figure S5

Reproducibility Checklist

## Data Availability

All datasets generated and analyzed during this study are included in this published article and its Supplementary Information files. Additional data are available from the corresponding author upon reasonable request.
