# Supplementary material for: SARS-CoV-2 infection induces persistent adipose tissue damage in aged golden Syrian hamsters
Source: Cell Death Dis. 2023 Feb 1;14(2):75. doi: 10.1038/s41419-023-05574-w (PMC9891765; doi:10.1038/s41419-023-05574-w)
Supplement: Supplementary file 1 — Supplementary Table S1 [file 41419_2023_5574_MOESM1_ESM.docx]

| **Gene** | **Forward primer sequence** | **Reverse primer sequence** |
| --- | --- | --- |
| *E SARS-CoV-2* | ACAGGTACGTTAATAGTTAATAGCGT | ATATTGCAGCAGTACGCACACA |
| *Isg15* | CTGGTGCCCCTGACTAACTC | CTGTCATTCCGCACCAGGAT |
| *Mx1* | GGTCTGGAACACTTGGGGAG | GGTCTGGAACACTTGGGGAG |
| *Fasn* | TCAAGACTACACACCACGGC | CACTGCCGTCTTCAGTCCTT |
| *Acacb* | GGACAGCTGAAGGCCCGAGA | CCAGCATGTGGCCTGGTGTG |
| *Scd1* | ACGGGAGAAGCAGAAGACCGT | CCTGGTAGCTGGGGTCGTGT |
| *Fads6* | GAAGGTGCCGTGCCTCAACC | AACGCTCGAGAGCGACCAGT |
| *Lipe* | GCATTGTGCCCTGCTCGGTT | CAGTGACGCAGAGGTTGCCG |
| *Pnpla2/3* | GCAGGGCTACAGAGACGGACT | TTTCCTGGGGGACAACCGGG |
| *Cpt1a* | CGAAAGTTGGACCGTGAGGA | ATCGTGGTAGAGCCAGACCT |
| *Acadvl* | GCAGGCGGGACGTAAGAGGA | CCGCCCCACACTCGGAGTTA |
| *Ifng* | TGTTGCTCTGCCTCACTCAGG | AAGACGAGGTCCCCTCCATTC |
| *Il1b* | GAAGTCAAAACCAAGGTGGAGTTT | TCTGCTTGAGAGGTGCTGATGT |
| *Actg1* | ACAGAGAGAAGATGACGCAGATAATG | GCCTGAATGGCCACGTACA |
| *GusB* | GTGCCAAGGACGCAGTGTTT | GGGCCTGCAGTGAGGTAGTTC |

**Supplementary Table S1. Sequences of the forward and reverse primers.** Primers were designed using the Primer Express^TM^ v3 software. *E SARS-CoV-2*: SARS-CoV-2 envelope protein, *Isg15*: interferon (IFN)-stimulated gene (ISG) 15, *Mx1*: IFN-induced GTP-binding protein, *Fasn*: fatty acid synthase, *Acacb*: acetyl-CoA carboxylase beta, *Scd1*: stearoyl-CoA desaturase 1, *Fads6*: fatty acid desaturase 6, *Lipe*: hormone sensitive lipase E, *Pnpla2/3*: adipose triglyceride lipase, *Cpt1a*: carnitine palmitoyl transferase 1A, *Acadvl*: very long-chain acyl-CoA dehydrogenase, *Ifng*: interferon gamma (γ), *Il1b*: interleukin-1 beta (β), *Actg1*: gamma-actin, and *GusB*: glucuronidase beta.
