# Supplementary material for: SARS-CoV-2 infection induces persistent adipose tissue damage in aged golden Syrian hamsters
Source: Cell Death Dis. 2023 Feb 1;14(2):75. doi: 10.1038/s41419-023-05574-w (PMC9891765; doi:10.1038/s41419-023-05574-w)
Supplement: Supplementary file 2 — Supplementary Table S2 [file 41419_2023_5574_MOESM2_ESM.docx]

|  | **#** | **Lipids** |
| --- | --- | --- |
| ↑ Young adults | 27 | TG(18 :3_36 :2), TG(18 :1_36 :4), TG(18 :2_33 :2), TG(18 :0_36 :4) TG(18 :1_36 :5), TG(18 :2_36 :5), TG(16 :0_36 :5), TG(18 :3_36 :3) TG(18 :2_34 :3), TG(18 :3_34 :2), TG(18 :2_33 :1), TG(18 :3_36 :4) TG(18 :0_36 :5), TG(17 :2_36 :4), TG(17 :1_36 :4), TG(18 :2_36 :2) TG(20 :2_34 :2), TG(18 :2_36 :3), TG(16 :0_36 :4), TG(18 :2_35 :1) CE(20 :1), TG(18 :2_34 :2), TG(22 :6_34 :2), TG(18 :2_35 :2), TG(18 :2_38 :5), TG(17 :0_36 :4), TG(16 :0_37 :3) |
| ↓ Young adults | 39 | TG(16 :0_33 :1), TG(16 :0_34 :2), TG(16 :1_34 :1), TG(14 :0_34 :3) TG(17 :0_34 :1), TG(16 :1_36 :1), TG(16 :0_32 :2), AA, CE(18 :2) DG(18 :1_18 :1), CE(20 :5), CE(16 :1), TG(18 :1_32 :0), FA(18 :2) TG(16 :0_34 :1), TG(18 :0_32 :1), TG(14 :0_35 :2), TG(18 :1_32 :1) TG(18 :1_30 :0), TG(18 :1_30 :1), TG(14 :0_34 :1), TG(14 :0_34 :2) TG(18 :1_33 :0), TG(16 :1_34 :0), CE(20 :4), TG(14 :0_36 :1), DHA TG(16 :1_32 :1), TG(16 :1_32 :0), TG(16 :0_32 :0), TG(16 :0_35 :1) TG(16 :0_32 :1), CE(18 :1), DG(14 :0_36 :2), TG(18 :0_32 :0) TG(14 :0_34 :0), TG(16 :0_34 :0), DG(16 :0_18 :1), TG(16 :1_32 :2) |
| ↓ Aged adults | 96 | TG(20 :5_34 :1), TG(18 :1_34 :3), TG(18 :3_34 :0), TG(18 :3_32 :0) TG(16 :0_35 :2), TG(18 :2_36 :1), TG(16 :1_34 :3), TG(18 :2_31 :0) TG(18 :1_32 :2), TG(14 :0_36 :2), TG(18 :1_33 :1), TG(16 :0_35 :3) TG(17 :0_34 :2), TG(18 :0_34 :2), TG(18 :2_30 :0), TG(18 :1_36 :1) TG(18 :1_34 :1), TG(16 :1_34 :2), TG(14 :0_36 :3), TG(18 :0_36 :2) FA(20 :2), TG(20 :1_32 :1), TG(18 :0_34 :3), TG(18 :3_36 :1) TG(14 :0_36 :4), TG(16 :0_32 :3), CE(20 :3), TG(16 :0_34 :4) TG(18 :1_31 :0), TG(18 :0_32 :2), TG(16 :1_33 :1), TG(16 :1_36 :4) TG(16 :0_34 :3), TG(17 :1_34 :1), FA(18 :1), TG(20 :2_32 :1) TG(18 :2_30 :1), TG(18 :3_32 :1), TG(18 :2_32 :2), TG(18 :2_33 :0) TG(17 :1_34 :2), TG(18 :1_34 :4), TG(16 :0_33 :2), TG(18 :2_32 :1) TG(18 :1_32 :3), TG(18 :2_32 :0), TG(18 :0_36 :1), TG(18 :2_34 :0) TG(18 :3_34 :1), TG(16 :0_38 :1), TG(16 :0_36 :2), TG(18 :3_36 :2) TG(18 :1_36 :4), TG(18 :2_33 :2), TG(18 :0_36 :4), TG(18 :1_36 :5) TG(18 :2_36 :5), TG(16 :0_36 :5), TG(18 :3_36 :3), TG(18 :2_34 :3) TG(18 :3_34 :2), TG(18 :2_33 :1), TG(18 :3_36 :4), TG(18 :0_36 :5) TG(16 :0_33 :1), TG(16 :0_34 :2), TG(16 :1_34 :1), TG(14 :0_34 :3) TG(17 :0_34 :1), TG(16 :1_36 :1), TG(16 :0_32 :2), AA, CE(18 :2) DG(18 :1_18 :1), CE(20 :5), CE(16 :1), TG(18 :1_32 :0), FA(18 :2) TG(16 :0_34 :1), TG(18 :0_32 :1), TG(14 :0_35 :2), TG(18 :1_32 :1) TG(18 :1_30 :0), TG(18 :1_30 :1), TG(14 :0_34 :1), TG(14 :0_34 :2) TG(18 :1_33 :0), TG(16 :1_34 :0), CE(20 :4), TG(14 :0_36 :1), DHA TG(16 :1_32 :1), TG(16 :1_32 :0), TG(16 :0_32 :0), TG(16 :0_35 :1) TG(16 :0_32 :1) |

**Supplementary Table S2.** **Differentially abundant plasma lipids in the day-7-post-SARS-CoV-2-infection condition vs. the mock condition in young adult and aged golden hamsters.** Lipids significantly increased (n=27) or decreased (n=39) upon SARS-CoV-2 infection in young adult hamsters, and lipids significantly decreased (n=96) upon SARS-CoV-2 infection in aged hamsters. Fold change>1.5, *p*<0.05.
