# Supplementary material for: SARS-CoV-2 infection induces persistent adipose tissue damage in aged golden Syrian hamsters
Source: Cell Death Dis. 2023 Feb 1;14(2):75. doi: 10.1038/s41419-023-05574-w (PMC9891765; doi:10.1038/s41419-023-05574-w)
Supplement: Supplementary file 3 — Legends to Supplementary Figures [file 41419_2023_5574_MOESM3_ESM.docx]

**LEGENDS TO SUPPLEMENTARY FIGURES**

**Supplementary Figure S1. Study design, including number of animals assigned per group.** Young adult (2-month-old) and aged adult (22-month-old) male golden (Syrian) hamsters were challenged either with 2x10^4^ TCID_50_ of SARS-CoV-2 or with DMEM (mock) by the intranasal route. SARS-CoV-2-infected animals were daily recorded for body weight from day 0 to 22 post-infection. Lungs, subcutaneous (inguinal) adipose tissue (SCAT),visceral (epidydimal) adipose tissue (VAT) and blood from mock-infected and SARS-CoV-2-infected (7 days post-infection (dpi) and 22 dpi) were collected for gene expression, histology, quantitative histomorphometry, immunohistochemistry, and plasma metabolomic analyses. The numbers of animals per group are indicated in blue.

Created with BioRender.com.

**Supplementary Figure S2. SARS-CoV-2 infection leads to decreased absolute and relative WAT amounts.** **A** Absolute and, **B** relative masses of (inguinal) SCAT and (epididymal) VAT of mock-treated and SARS-CoV-2-infected young adult and aged hamsters. Data are expressed as the mean ± SEM, and individual replicates are shown (mock and 7 dpi: n=6 animals per group, 22 dpi: n=6 young adults and n=3 aged adults). Groups were compared in a two-sided Mann-Whitney test ; # indicates the *p* values for the comparison of young adults and aged adults (the effect of age: ^#^ *p*<0.05 and ^##^ *p*<0.01) and * indicates the *p* values for the comparison of mock-treated and SARS-CoV-2-infected groups (the effect of infection: ^*^*p*<0.05 and ^**^ *p*<0.01). For intergroup differences, the threshold for statistical significance was set to *p*<0.05.

**Supplementary Figure S3. Aging has a regional impact on adipose tissue inflammation.** **A** mRNA expression levels (RT-qPCR assay) of *Fasn* and *Acacb* (involved in fatty acid synthesis), *Scd1* and*Fasd6* (involved in fatty acid desaturation), *Lipe* and *Pnpla2/3* (involved in triglyceride degradation), and *Cpt1a* and *Acadvl* (involved in fatty acid oxidation) in the (epididymal) VAT of mock-treated young adult and aged hamsters. **B** mRNA expression levels (RT-qPCR assay) of the inflammatory genes *Ifng* and *Il1b* in the VAT of mock-treated young adult and aged hamsters. For **A** and **B**, relative expression is presented as 2^−ΔΔCT^ (housekeeping gene=*GusB* coding for glucuronidase beta). Data are expressed as the mean ± SEM, and individual replicates are shown (n=6 animals per group). Groups were compared in a two-sided Mann-Whitney test. For intergroup differences, the threshold for statistical significance was set to *p*<0.05.

**Supplementary Figure S4. SARS-CoV-2 infection alters SCAT and VAT lipid metabolism.** mRNA expression levels (RT-qPCR assay) of *Fasn*, *Acacb*, *Scd1*, *Fasd6*, *Lipe*, *Pnpla2/3*, *Cpt1a* and *Acadvl* in **A** the (inguinal) SCAT, and **B** the (epididymal) VAT of young adult and aged hamsters, at 0 (mock), 7 and 22 dpi.

For **A** and **B**, relative expression is presented as 2^−ΔΔCT^ (housekeeping gene: *GusB*, coding for glucuronidase beta). Data are expressed as the mean ± SEM, and individual replicates are shown (n=6 animals per group ; except for 22 dpi: aged adult hamsters n=3).

Groups were compared in a two-sided Mann-Whitney test ; # indicates the *p* values for the comparison of young adults and aged adults (the effect of age: ^#^ *p*<0.05 and ^##^ *p*<0.01), and * indicates the *p* values for the comparison of mock-treated and SARS-CoV-2 infected groups (the effect of infection: ^*^*p*<0.05 and ^**^ *p*<0.01). For intergroup differences, the threshold for statistical significance was set to *p*<0.05.

**Supplementary Figure S5. Impact of SARS-CoV-2 infection on VAT. A** Representative microscopy images of the (epididymal) VAT of young adult and aged hamsters at 0 (mock), 7 and 22 dpi (H&E staining). **B** Representative IHC staining for perilipin-1 in the VAT of young adult and aged hamsters at 0 (mock), 7 and 22 dpi identified live (stained) adipocytes. Note that no perilipin-null (dead) adipocytes were observed. **C** Representative HIC staining for SARS-CoV-2 spike protein (brown precipitate) around conventional CLSs, in the (inguinal) SCAT of 7-dpi-infected aged hamsters. For **A** to **C**, scale bars=100 μm. For **B** and **C**, slides were counterstained with Mayer’s hematoxylin.
