# Supplementary figures and images for: SARS-CoV-2 infection induces persistent adipose tissue damage in aged golden Syrian hamsters
Source: Cell Death Dis. 2023 Feb 1;14(2):75. doi: 10.1038/s41419-023-05574-w (PMC9891765; doi:10.1038/s41419-023-05574-w)

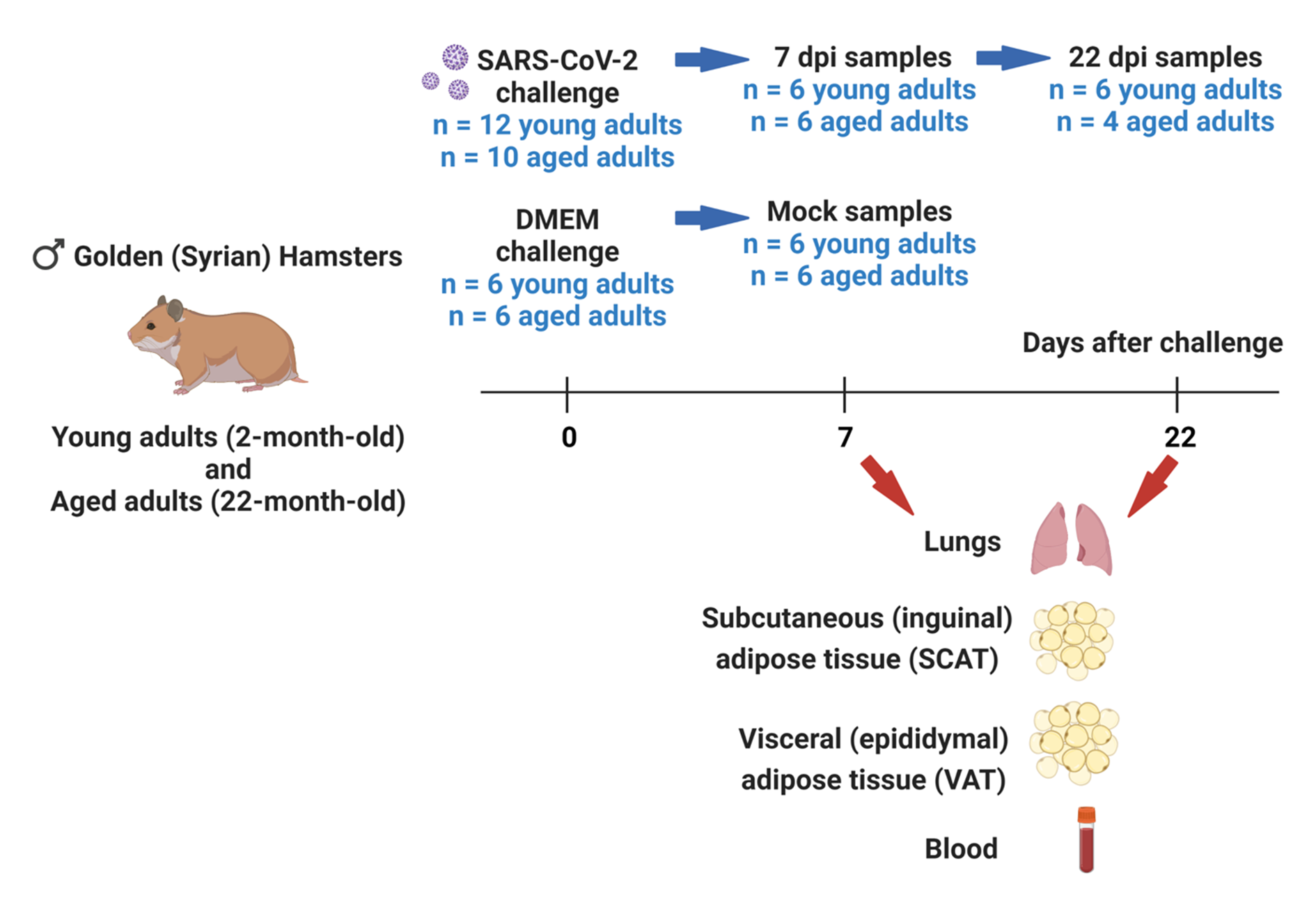

Supplement: Supplementary file 4 — Supplementary Figure S1 [file 41419_2023_5574_MOESM4_ESM.tif]

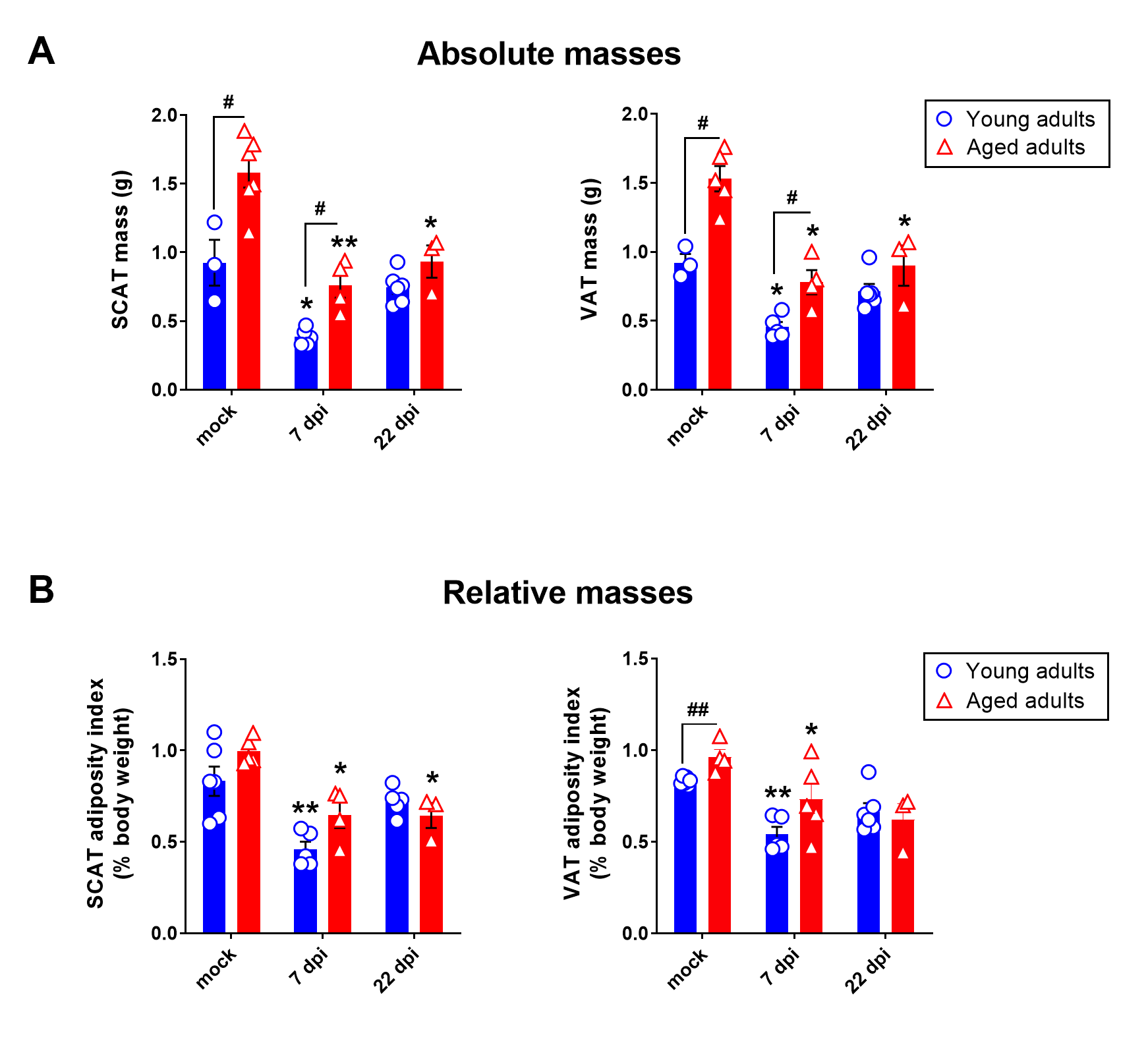

Supplement: Supplementary file 5 — Supplementary Figure S2 [file 41419_2023_5574_MOESM5_ESM.tif]

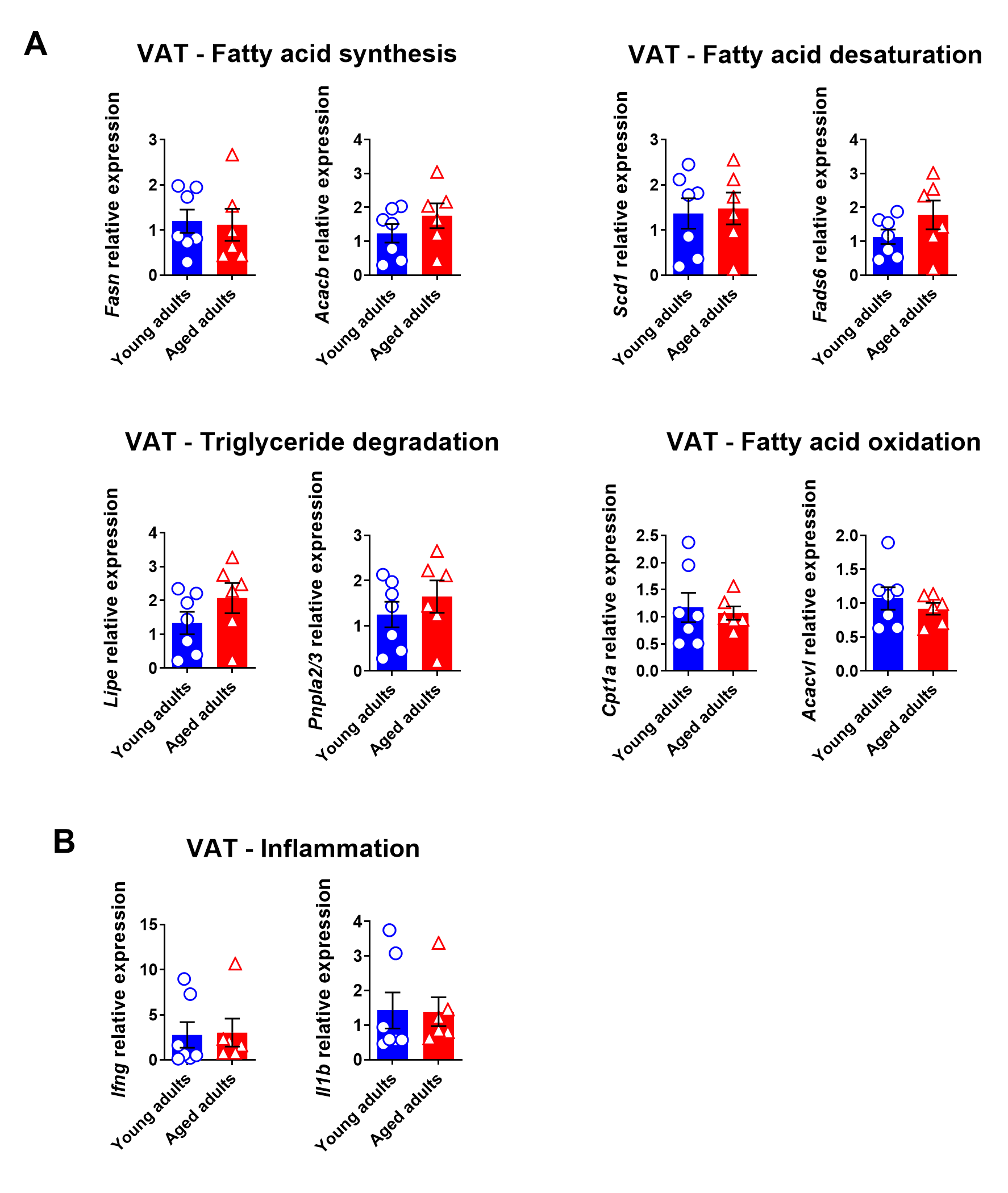

Supplement: Supplementary file 6 — Supplementary Figure S3 [file 41419_2023_5574_MOESM6_ESM.tif]

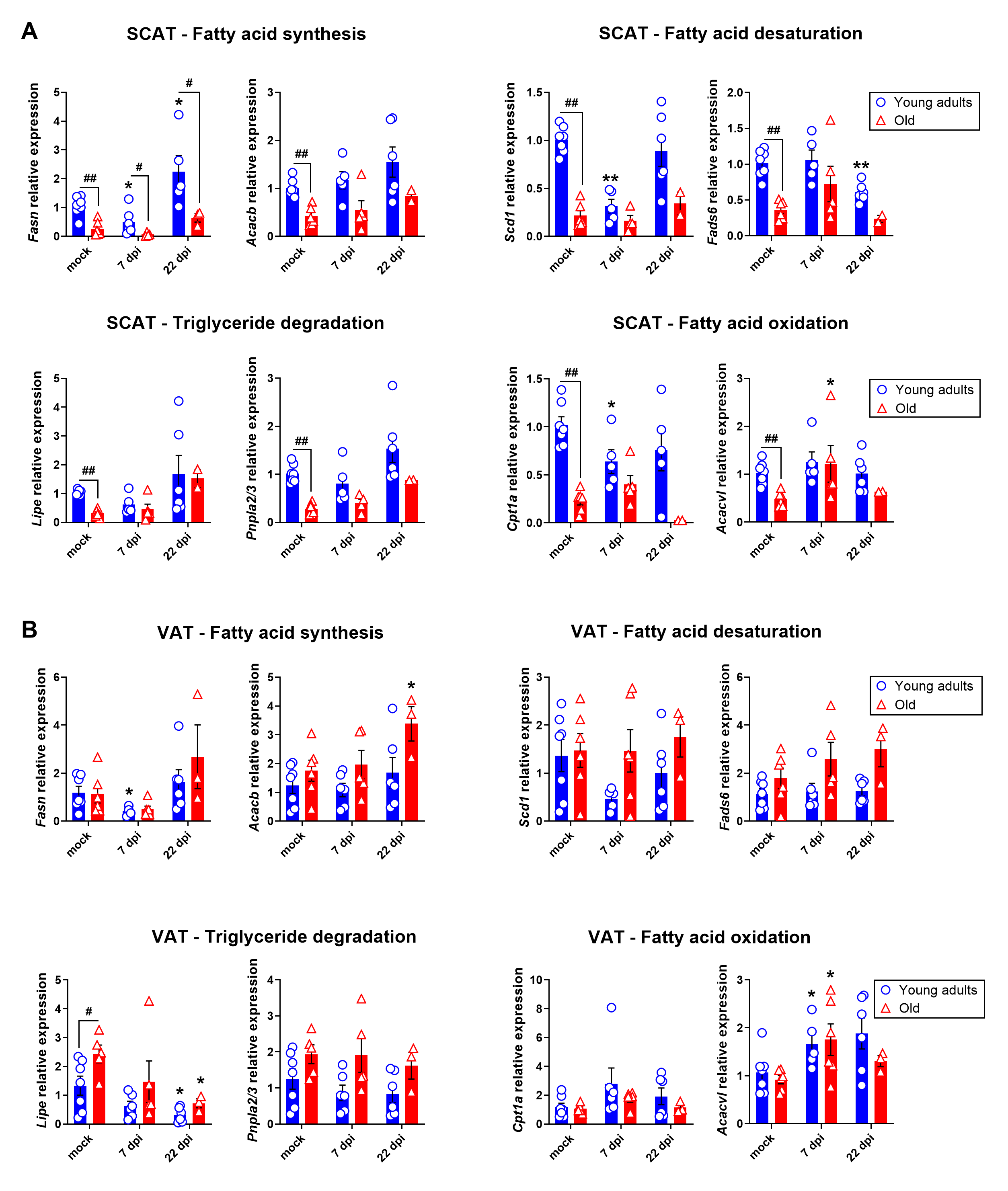

Supplement: Supplementary file 7 — Supplementary Figure S4 [file 41419_2023_5574_MOESM7_ESM.tif]

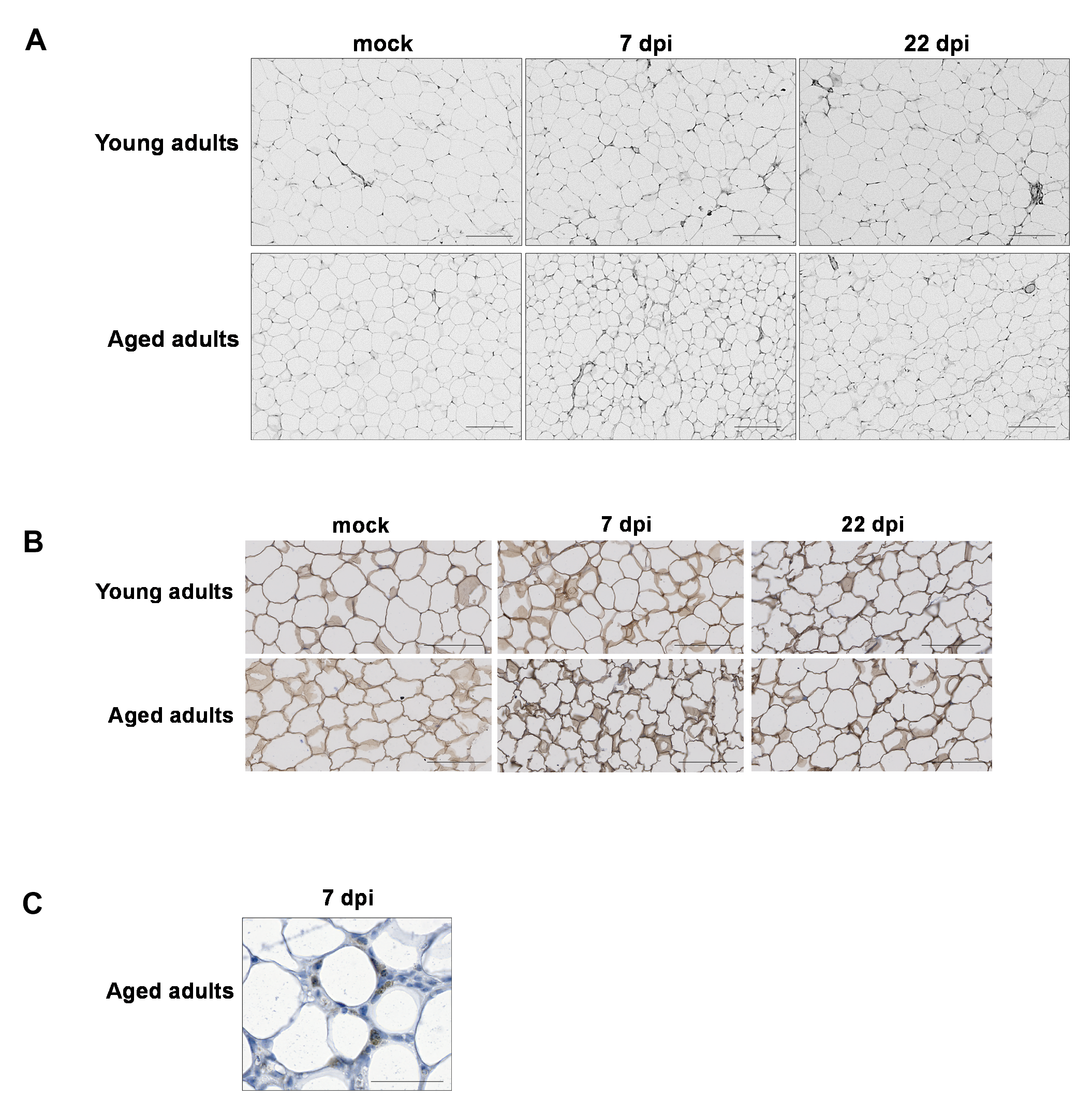

Supplement: Supplementary file 8 — Supplementary Figure S5 [file 41419_2023_5574_MOESM8_ESM.tif]
